# Fibroblast growth factor receptor inhibitors mitigate the neuropathogenicity of *Borrelia burgdorferi* or its remnants *ex vivo*

**DOI:** 10.3389/fimmu.2024.1327416

**Published:** 2024-04-04

**Authors:** Geetha Parthasarathy

**Affiliations:** Division of Immunology, Tulane National Primate Research Center, Tulane University, Covington, LA, United States

**Keywords:** Lyme neuroborreliosis, *B. burgdorferi*, rhesus frontal cortex, rhesus DRG, FGFR, neuroinflammation

## Abstract

In previous studies, we showed that fibroblast growth factor receptors (FGFRs) contribute to inflammatory mediator output from primary rhesus microglia in response to live *Borrelia burgdorferi*. We also demonstrated that non-viable *B. burgdorferi* can be as pathogenic as live bacteria, if not more so, in both CNS and PNS tissues. In this study we assessed the effect of live and non-viable *B. burgdorferi* in inducing FGFR expression from rhesus frontal cortex (FC) and dorsal root ganglion (DRG) tissue explants as well as their neuronal/astrocyte localization. Specific FGFR inhibitors were also tested for their ability to attenuate inflammatory output and apoptosis in response to either live or non-viable organisms. Results show that in the FC, FGFR2 was the most abundantly expressed receptor followed by FGFR3 and FGFR1. Non-viable *B. burgdorferi* significantly upregulated FGFR3 more often than live bacteria, while the latter had a similar effect on FGFR1, although both treatments did affect the expressions of both receptors. FGFR2 was the least modulated in the FC tissues by the two treatments. FGFR1 expression was more prevalent in astrocytes while FGFR2 and FGFR3 showed higher expression in neurons. In the DRG, all three receptor expressions were also seen, but could not be distinguished from medium controls by immunofluorescence. Inhibition of FGFR1 by PD166866 downregulated both inflammation and apoptosis in both FC and DRG in response to either treatment in all the tissues tested. Inhibition of FGFR1-3 by AZD4547 similarly downregulated both inflammation and apoptosis in both FC and DRG in response to live bacteria, while with sonicated remnants, this effect was seen in one of the two FC tissues and 2 of 3 DRG tissues tested. CCL2 and IL-6 were the most downregulated mediators in the FC, while in the DRG it was CXCL8 and IL-6 in response to FGFR inhibition. Downregulation of at least two of these three mediators was observed to downregulate apoptosis levels in general. We show here that FGFR inhibition can be an effective anti-inflammatory treatment in antibiotic refractive neurological Lyme. Alternatively, two biologics may be needed to effectively curb neuroinflammation and pathology in the CNS and PNS.

## Introduction

Lyme disease (LD), caused by the bacterium *Borrelia burgdorferi* is the leading tick-borne illness in the United States, accounting for nearly 70% of all tick-borne infections (1). Recent estimates indicate that the case load of LD is ~476,000 per year (2), up from the ~5000 cases in the early to mid-eighties (3), indicating a significant emergence (or diagnosis) of this disease in recent years. As a bacterial infection, LD is treated with antibiotics. However, approximately 10- 35% of patients treated for LD have various lingering ailments, collectively called post-treatment Lyme disease syndrome or PTLDS (4, 5). These symptoms can range from fatigue, cognitive issues, memory loss, neuropathy, joint pain, musculoskeletal pain, to sleep issues, nausea, depression, and others (6, 7). Persistent neuroinflammation may be a likely source of such symptoms as many studies have shown a link between neuroinflammatory processes and cognitive defects, fatigue, neuropathy, autoimmune diseases, and others (8–10). In support of this, PET scans of PTLDS patients show likely glial activation in the brain, several months post-treatment (11), indicating on-going neuroinflammation.

The cause of such persistent neuroinflammation is not clear and may be effected by live residual bacteria impervious or inaccessible to antibiotics (12, 13), anti-*B. burgdorferi* antibodies that cross react with nervous tissues, leading to glial activation (14–18), microbiome changes that affect the CNS through the gut-brain axis (19, 20) and unresolved coinfections (21). In addition to these hypotheses, residual antigens after antibiotic treatment might also elicit (neuro)inflammation. Persisting antigenic fragments/DNA post antibiotic treatment in both mice and humans with Lyme Borreliosis have been demonstrated (22–25). Intraperitoneal inoculation of *Escherichia coli* LPS in mice has been shown to induce inflammatory mediators in the CNS that persist for ten months (26). In this study, peripheral inoculation of LPS was shown to chronically activate microglia in the mouse brain, along with loss of neurons. In support of the “antigenic fragments” hypothesis, we have shown that non-viable *B. burgdorferi* can induce neuroinflammatory mediators from human oligodendrocytes, rhesus CNS and PNS tissues along with concomitant apoptosis (27, 28). In recent studies, we have shown that the fibroblast growth factor receptors (FGFRs) are activated in response to live *B. burgdorferi* in primary rhesus microglia and showed that these receptors are proinflammatory in response to both live and sonicated Lyme disease bacterium (29).

However, as *in vitro* studies on a single glial cell may not reflect the overall inflammatory output in complex nervous tissues, in the current study, the role of FGFR receptors in primary rhesus frontal cortex (FC) and dorsal root ganglion (DRG) tissues in response to both live and non-viable *B. burgdorferi* was explored. In addition, the ability of specific FGFR inhibitors to curb both neuroinflammation and apoptosis as mediated by either treatment was also assessed. The results show that like the *in vitro* data on primary microglia, FGFRs are pathogenic factors in nervous tissues as well. The study also shows that inhibiting FGFRs may be an effective anti-inflammatory therapy for persistent neuroinflammation even in antibiotic refractive conditions.

## Materials and methods

### Bacterial culture

*B. burgdorferi* strain B31, clone 5A19 was used throughout this study, and cultured according to previously published protocols (28). For experimentation, bacterial concentration was determined using a dark field microscope. The required number of bacteria was harvested by centrifugation at 2095 x g for 30 minutes at room temperature, without brakes. Bacteria were resuspended in RPMI 1640 medium (BioWhittaker, Fisher Scientific, Waltham, MA) supplemented with 10% fetal bovine serum (FBS) (Hyclone, GE Lifesciences, Pittsburgh, PA) to the original concentration initially and then diluted as required in the same medium. Non-viable *B. burgdorferi* were obtained by sonication according to previous protocols (27).

### *Ex vivo* assays

CNS (frontal cortex) and PNS (DRG) tissues from uninoculated rhesus macaques (*Macaca mulatta*) were used to test the efficacy of FGFR inhibitors in downregulating neuroinflammation induced by live *B. burgdorferi* or its remnants. Tissues were obtained from animals that were euthanized from the breeding colony due to persistent diarrhea, or injury. All euthanasia procedures were performed by veterinarians according to Tulane Institutional Animal Care and Use Committee approved protocols. The animals sourced for the tissues are listed in **Table 1**. CNS tissues were sliced with a tissue slicer (Ted Pella Inc., Redding, CA) to approximately 2 mm thickness, while the smaller DRG tissues were cut with a scalpel. Freshly cut sections were transferred to 12-well plates containing RPM1 1640 medium with 10% FBS initially and then replaced with same medium containing 1 x 10^7^/mL of live *B. burgdorferi* or its sonicated equivalent. Various FGFR inhibitors (FGFR1 inhibitor PD166866 (Millipore Sigma, Burlington, MA); FGFR1-3 inhibitor (and likely FGFR4) AZD4547 (MedChem Express, Monmouth Junction, NJ)) or DMSO was then added and incubated for 4h at 37°C, 5% CO_2_. For a given tissue, specificity of treatments or number of doses tested depended on the tissue availability. For a given inhibitor, tissues from the same animal tested with both live and sonicated remnants often had a common medium control. Tissue sections with medium alone, containing DMSO solvent control was used as negative controls, while sections with live bacteria/DMSO or sonicated *B. burgdorferi*/DMSO were considered as positive controls for inflammatory mediator/apoptosis induction. Supernatants were collected and stored at -20 °C until analysis, while the tissues were fixed as described previously (28).

**Table 1:** Age, sex and strain of the animal tissues used in this study.

Animal Number	Age (years)	Sex, Strain
**1**	10.68	Male, Indian rhesus
**2**	3.08	Male, Indian rhesus
**3**	1.50	Male, Indian rhesus
**4**	10.8	Female, Indian rhesus
**5**	2.26	Male, Indian rhesus
**6**	8.16	Female, Indian rhesus
**7**	21.98	Female, Indian rhesus
**8**	8.95	Female, Chinese rhesus
**9**	0.77	Female, Indian rhesus
**10**	0.72	Female, Indian rhesus
**11**	21.62	Male, Indian rhesus

### Multiplex assays

Supernatants from the *ex vivo* explants were analyzed for IL-6, CXCL8 and CCL2 by custom multiplex (Procartaplex) kits or individual kits (Life technology Corporation (Grand Island, NY). Multiplex assays were carried out at the Pathogen Detection and Quantification Core, Tulane National Primate Research Center according to the manufacturer’s protocols. The results were graphed using GraphPad Prism Software version 9.

### Immunofluorescence

Immunostaining of specific FGFRs was performed as described previously, in sections without DMSO, using archival tissues (28). Briefly, 7-10 µm thick cryosections were permeabilized in Phosphate buffered saline (PBS) containing 0.1% Triton-X-100 for 15 minutes, on a shaking platform. Tissues were then blocked in PBS with 10% Normal goat serum for 1h to reduce non-specific binding and background staining. Sections were then probed with various primary antibodies for 1h at room temperature, followed by corresponding secondary antibodies (1:800-1:1000) conjugated to fluorochromes, for 45 minutes to an hour. Slides were then mounted with an anti-quenching reagent, cover slipped and visualized for microscopy. The following primary antibodies were used: rabbit anti-human FGFR1 (1:100, MyBioSource, San Diego, CA, or Abcam, Waltham, MA), rabbit anti-human FGFR2 (1:100, ThermoFisher Scientific, Waltham, MA); rabbit anti-human FGFR3 (1:100, ThermoFisher Scientific); mouse anti-GFAP-cy3 (1:200-1:500, Sigma Aldrich, St. Louis, MO), mouse anti-MAP2 (1:50, Sigma Aldrich); anti-S100 (1:500, Sigma Aldrich) and mouse anti- NeuN (1:10, Millipore Sigma, Burlington, MA), or DAPI (1:5000, Millipore Sigma).

### Terminal Deoxynucleotidyl Transferase dUTP Nick-End Labeling (TUNEL) assay

The effect of FGFR inhibitors on apoptosis as induced by *B. burgdorferi* treatments was analyzed by the TUNEL assay, according to the manufacturer’s protocols (EMD Millipore Apoptag Fluorescein kit). Cell specific staining, if required, was performed prior to TUNEL staining according to prior published protocols (28, 30). As with immunofluorescence, at the end of the assay, slides were mounted, and covered with coverslip prior to microscopy.

### Microscopy

Immunofluorescence was visualized with a Nikon Ti2- E fluorescent microscope (Nikon, Melville, NY), equipped with NIS- Elements software (Nikon Instruments). Leica DMRE fluorescent microscope (Leica microsystems, Buffalo Grove-IL) and Lumecor SOLA GUI software (Lumencor, Beaverton-OR) were also used, wherein images were captured with Nuance Multispectral Imaging System (CRi, PerkinElmer, Waltham- MA). Confocal microscopy images, when needed, were captured with Leica TCS SP2 confocal microscope (Leica microsystems, Buffalo Grove, IL). Images were assembled with Adobe^®^ Photoshop CS6 software. FGFR expression levels in the FC were semi-quantitated by counting FGFR positive cells over 5-10 frames to obtain an average. Similarly, apoptotic nuclei per frame was semi-quantitated over 2-10 frames for DRG sections and 10-40 frames for FC sections. Results were graphed in Microsoft Excel^®^ for FGFR expression and in GraphPad Prism for apoptotic assays. Final data was assembled in Microsoft PowerPoint^®^, Adobe^®^ acrobat and Adobe^®^ Photoshop.

### Statistics

Statistical significance was determined using a student’s t-test (2-tailed) for FGFR expression studies, and one way ANOVA/Dunnett’s *post hoc* for all others. All analysis was in duplicate. Values ≤ 0.05 were considered statistically significant.

## Results

### FGFRs are expressed in response to live or non-viable *B. burgdorferi* in the frontal cortex

**Figure 1:**
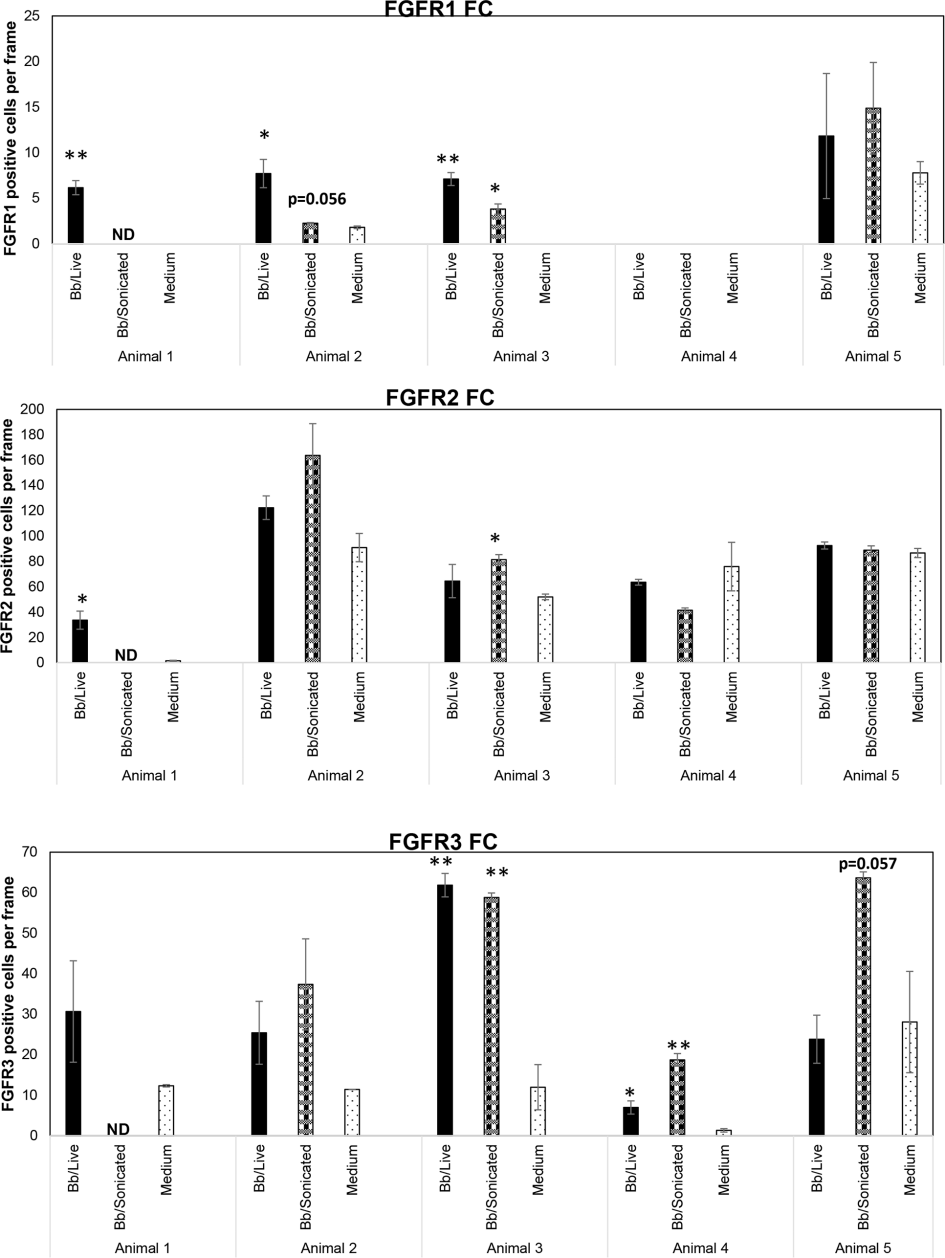
Expression levels of FGFRs from rhesus frontal cortex (FC) in response to live and non-viable *B burgdorferi*. FC tissue explants exposed to either live or sonicated *B burgdorferi* for 4 hrs. were probed for FGFR1-3 expression levels by immunofluorescence. Tissue explants exposed to medium alone were used as controls. FGFR positive cells per frame were quantified and graphed. Bars represent standard deviation. * p < 0.05; ** p < 0.01. ND- not determined.

**Figure 2:**
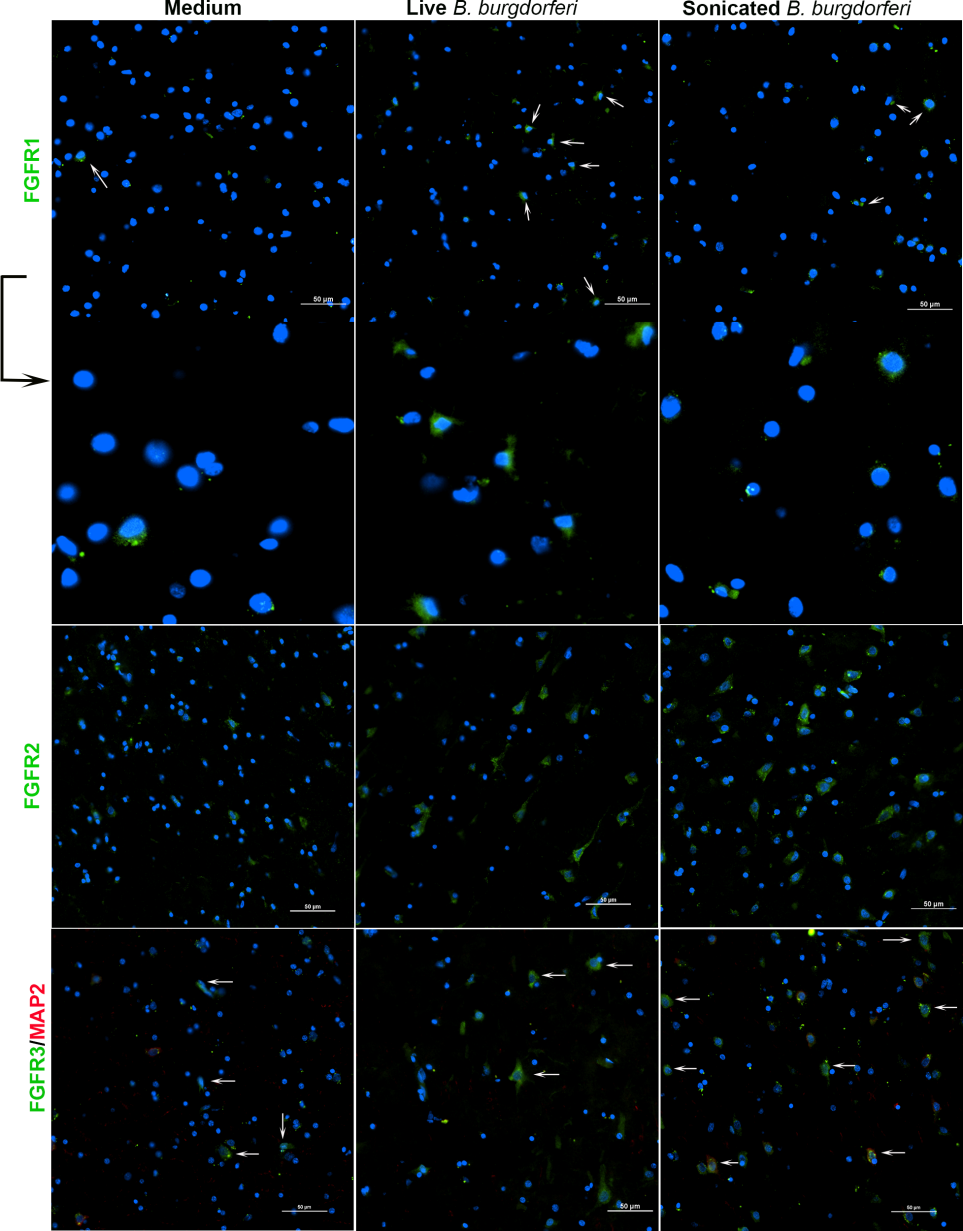
FGFR 1-3 expression from rhesus frontal cortex (FC) in response to live and non-viable *B burgdorferi*. FC tissue explants were probed for FGFR1-3 expression by immunofluorescence as described in methods. Respective FGFR expression is shown in green while the nuclear stain is in blue. Neuronal MAP2 staining, if performed, is in red. White arrows indicate some of the cells expressing FGFRs. The panels below FGFR1 show the magnification of cells indicated by the arrows above. FGFR1 staining from Animal 2 is shown, while FGFR2 and FGFR3 are from Animal 5.

FGFR 1-3 expression in the FC tissue explants without DMSO was analyzed. The results are shown in **Figures 1**, **2**.

Considering the FGFR levels in the medium only controls, FGFR2 was the most abundant receptor, followed by FGFR3 and then FGFR1 (**Figure 1**). Exposure to bacterial treatments modulated these levels depending on the animal. In response to live bacteria, FGFR1 expression was significantly upregulated in 3/5 tissues tested, while sonicated bacterial remnants induced significant upregulation in 1/4 tissues tested. One animal tissue failed to induce FGFR1 with either treatment. FGFR2 levels were significantly increased in 1/5 tissues and 1/4 tissues by live bacteria and non-viable *B. burgdorferi* remnants respectively. FGFR3 expression was significantly upregulated in 2/5 tissues in response to live bacteria, while *B. burgdorferi* remnants significantly induced this receptor expression in 2/4 tissues. In summary, FGFR1 was more significantly upregulated by live bacteria, while non-viable remnants had a greater impact on FGFR3 levels. FGFR2 was the least modulated in terms of significant changes (in cell numbers). However, since biological effects can be induced by increased levels of expression, with or without statistical significance, overall changes in mean expression levels were then examined.

Cumulatively, higher mean FGFR1 levels were induced by live bacteria in 4/5 tissues, and by sonicated remnants in 3/4 FC tissues. Similarly, mean FGFR2 levels were noticeably higher in 3/5 in response to live bacteria, and 2/4 in response to remnants. Mean FGFR3 levels on the other hand, were higher in response to remnants in all 4/4 tissues tested, while they were higher than in medium controls in 4/5 tissues in response to live *B. burgdorferi*. These data indicate that FGFR1-3 levels are modulated by exposure to the Lyme disease bacterium or its remnants.

While **Figure 1** shows the number of cells expressing a specific FGFR, **Figure 2** shows staining patterns and intensities. At the same magnification, FGFR2 and FGFR3 had higher visibility compared to FGFR1, indicating differential intensity or differential cell expression. A negative control slide with the fluorescent secondary antibody alone is shown in **Supplementary Figure 1** (SF1). Intensity differences between medium and treatment groups could also be seen qualitatively, indicating expression changes can occur without an appreciable increase in cell numbers staining positive for receptors. A more precise quantification analysis awaits future availability of these scarce resources.

### FGFR expression has a predominant neuronal prevalence in the frontal cortex

**Figure 3:**
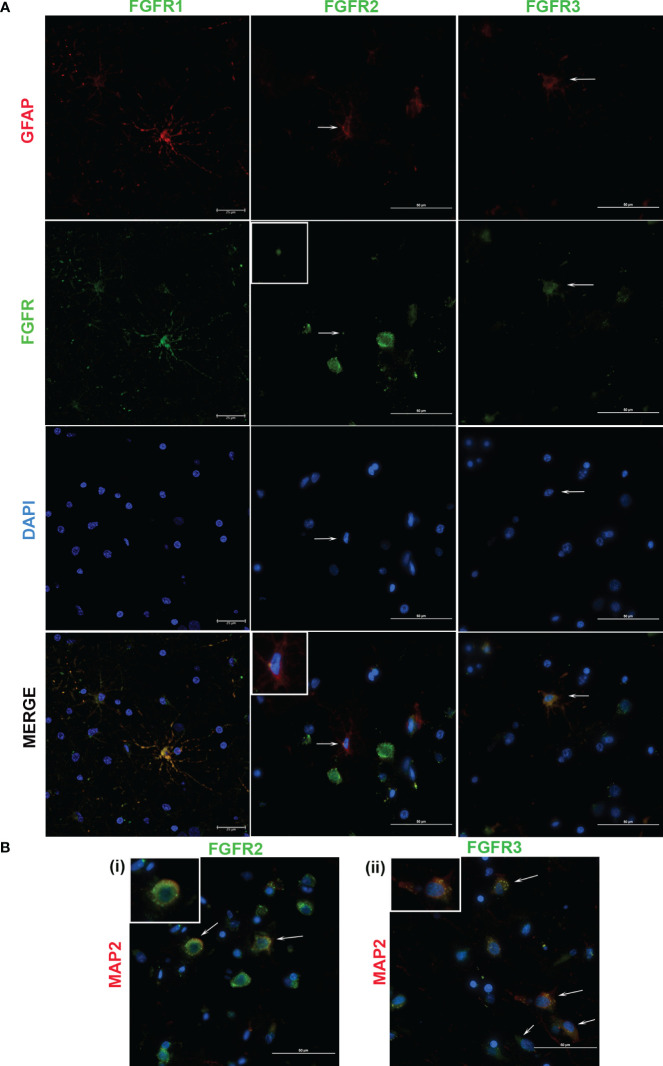
Cellular expression of FGFRs in FC in response to sonicated *B burgdorferi*. The cell types expressing specific FGFRs (green), were probed with cell specific and receptor specific antibodies. Astrocytic (GFAP, red) specific FGFR expression is shown in **(A)**. In FGFR2-related panels, the inset images show magnified regions indicated by the corresponding arrows. All the three FGFR expressions are from Animal 2. **(B)** shows neuronal expression (MAP2, red) of the indicated FGFRs. The inset images show magnification of one of the cells indicated by the arrows. FGFR2 staining **(i)** is from Animal 2, while FGFR3 **(ii)** is from Animal 5. In both **(A, B)**, nuclei are stained blue with DAPI. FGFR1 expression in **(A)** was captured by confocal microscopy, while the rest of the images were obtained by a fluorescent microscope.

**Figure 4:**
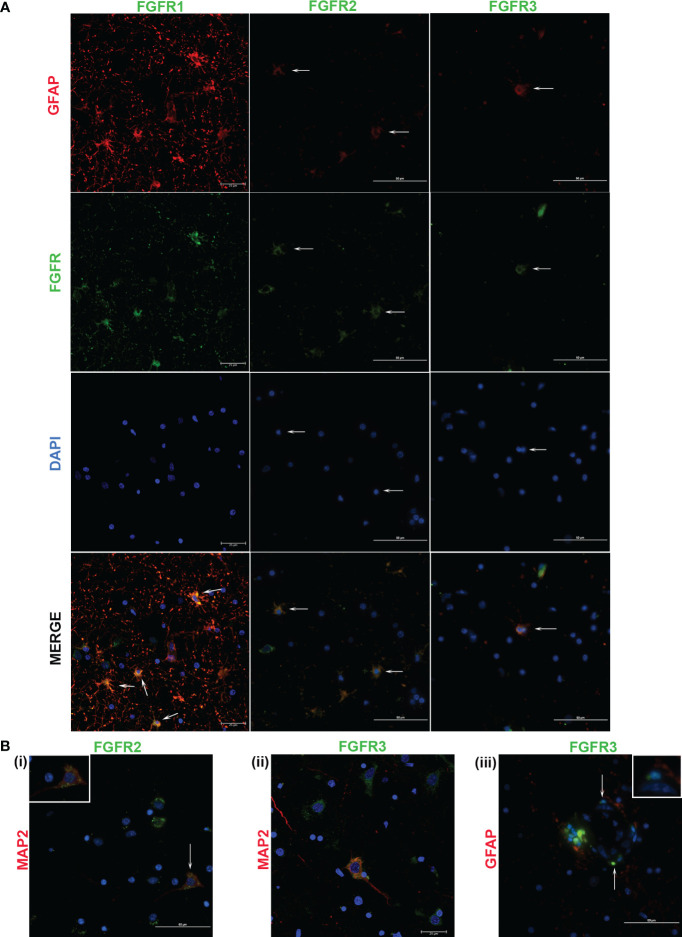
Cellular expression of FGFRs in FC in response to live *B burgdorferi*. **(A)** shows expression of the indicated FGFRs (green) in astrocytes (GFAP, red). FGFR1 and FGFR2 staining pattern was obtained from Animal 2, while FGFR3 was from Animal 3. In **(B)**, FGFR2 and FGFR3 expressions in MAP2 positive neurons (red) are shown in **(i)** and **(ii)** respectively. Inset in **(B (i))** shows the cell indicated by the corresponding arrow. In **(B (iii))**, cells in the presumed blood-brain barrier show positivity for FGFR3. Inset shows colocalization of the blue nuclear stain with the green FGFR3 resulting in cyan. All the fluorescent micrographs in **(B)** are from Animal 3. Except for FGFR1 panels and **Figure 4** **(B (ii))**, which show confocal microscopy images, all the images in **Figure 4** were obtained by a fluorescent microscope.

In previous studies, we showed that exposure of primary rhesus microglia to live *B. burgdorferi* upregulated the expression of all three FGFR receptors (29). FGFR1-3 expressions in MO3.13 human oligodendrocytes in response to live *B. burgdorferi* has also been examined (31). Therefore, in this study, the cell specific analysis of FGFRs in FC explants was confined to astrocytes (GFAP positive) and neurons (MAP2 positive) and is shown in **Figures 3**, **4**. (On a technical note, when the red and green pixels are equal in intensity, a yellow color is seen on colocalization. When the red intensity is predominant over green, an orange color is seen).

FGFR1 expression in FC tissues treated with live or sonicated remnants showed an astrocyte prevalence. A few non-GFAP cells exhibiting green FGFR1 signal were also seen (**Figure 4**, FGFR1 merge panel). Considering their large size, these are likely neurons.

In tissues treated with live bacteria or its sonicated remnants, FGFR2 expression, unlike FGFR1, was predominant in neurons (**Figures 3Bi**, **4Bi**). Of note, MAP2 staining did not stain all neurons uniformly, but, neurons can be distinguished by their large nucleus and a less intense DAPI staining pattern (**Supplementary Figure 2**) (SF2). A few astrocytes staining positive for FGFR2 were also seen with either treatment (**Figures 3A**, **4A**).

Similar to the FGFR2 pattern of expression, FGFR3 staining was also more prevalent in neurons in tissues exposed to either live or sonicated remnants (**Figures 3Bii**, **4Bii**). Again, like FGFR2 expression, a few astrocytes staining positive for FGFR3 can be seen (**Figures 3A**, **4A**). FGFR3 expression in the presumed blood brain barrier (BBB) areas were also noted (**Figure 4Biii**) indicating expression in likely astrocytic end feet areas or possibly in pericytes (inset). Similar expression of FGFR2 in BBB was also seen (not shown).

The colocalization patterns between cell markers and receptors also varied throughout. Although FGFRs are generally considered as surface receptors, their normal localization can vary depending on the cell type (29, 31). Therefore, colocalization signals of FGFRs and cell markers depend upon the receptor localization (extracellular, intracellular, or internalized (32)) and may account for the different patterns of cellular staining seen.

In summary, the data shows that FGFR1 is predominant in astrocytes while FGFR2 and FGFR3 in neurons in response to treatments- although both cells stain for all three receptors. But, how these individual cell types modulate specific receptors in response to specific bacterial treatment (in comparison to medium controls) could not be enumerated from immunofluorescence alone due to uneven staining of cells. Therefore, the modulation patterns in astrocytes or neurons alone in response to live bacteria or remnants remains to be examined by individual cell cultures, and perhaps ELISA in addition.

### FGFRs are also expressed in the dorsal root ganglion

FGFR1-3 expression levels in the DRG in response to live or sonicated *B. burgdorferi* were also investigated by immunofluorescence and are shown in **Supplementary Figure 3** (SF3). Unlike the FC, where modulation of these receptors was demonstrated, DRG sections did not show any appreciable differences between the medium controls and either treatment conditions, although all 3 receptor expressions were seen. It is likely that ELISA of individual cell types would be needed to delineate differences if any, in receptor expressions.

### FGFR1 inhibitor downregulates neuroinflammatory mediators in both the FC and DRG in response to either live *B. burgdorferi* or its remnants

The effect of FGFR1 inhibitor PD166866 on inflammatory mediator release from nervous tissues in response to either live or non-viable *B. burgdorferi* is shown in **Table 2** (and **Supplementary Figures 4, 5**). PD166866 concentrations used in FC were based on our previous study (29), while lower concentrations used in the DRG were based on its much smaller size.

**Table 2:** Fold^a^ downregulation in chemokines/cytokines and apoptosis in response to PD166866 FGFR1 inhibitor.

Tissue	Bacteria type	Animal #	Chemokines/Inhibitors	CCL2	CXCL8	IL-6	Apoptosis
**FC**	**Sonicated**	**#2**	10 µM PD166866	**4.88**	**4.92**	**5.22**	**1.42**
			5 µM PD166866	**3.13**	**4.04**	**2.58**	**1.67**
			1 µM PD166866	**1.26**	**1.77**	0.99	1.20
			
		**#5**	10 µM PD166866	**2.82**	1.23	**4.14**	**1.40**
			5 µM PD166866	**2.81**	0.98	**3.05**	**1.19**
			1 µM PD166866	**2.75**	***0.75***	**3.34**	**1.53**
			
	**Live**	**#3**	10 µM PD166866	**4.33**	**4.51**	2.44	**1.76**
			5 µM PD166866	**2.11**	**1.80**	0.66	**1.29**
			1 µM PD166866	**2.31**	**5.02**	0.96	**2.32**
			
		**#5**	10 µM PD166866	**2.80**	**2.03**	**2.38**	**1.80**
			1 µM PD166866	**2.51**	1.14	**1.86**	**2.61**
			
**DRG**	**Sonicated**	**#5**	1 µM PD166866	***0.90***	**1.39**	**2.01**	**1.26**
			500 nM PD166866	0.93	1.06	**2.26**	**1.77**
			100 nM PD166866	1.01	**2.59**	**3.31**	**1.80**
			
		**#6**	1 µM PD166866	**1.11**	**3.37**	**1.86**	**1.47**
			500 nM PD166866	1.03	**1.62**	1.16	**1.35**
			100 nM PD166866	***0.72***	**2.67**	**1.45**	**3.09**
			
	**Live**	**#5**	1 µM PD166866	**5.21**	**1.47**	**1.83**	ND
			500 nM PD166866	***0.58***	**1.92**	**2.96**	**2.39**
			100 nM PD166866	***0.55***	**3.27**	**3.18**	**1.62**
			
		**#6**	1 µM PD166866	**1.20**	1.15	**1.17**	**1.59**
			500 nM PD166866	**1.17**	0.85	**1.34**	**1.75**
			100 nM PD166866	0.97	**1.42**	**2.23**	**1.45**

a Fold change was calculated as inflammatory levels or apoptosis levels induced by Bb + DMSO group/Bb with treatment group. Numbers greater than 1 indicate downregulation of inflammatory mediators or apoptosis while numbers lower than one indicate an increase. When the downregulation is statistically significant, they are indicated in bold and underlined. When they are significantly increased, the fold change levels are bold and in italics. Calculated from **Supplementary Figures 4, 5** and **8**. ND, Not determined.

To evaluate the effect of FGFR inhibitors, several factors were taken into consideration, one being the variations between tissue slices. FC is not a uniform structure, consisting of multiple layers and variations in cell types within layers (33–35). As explants were cut from a single block of FC tissue from each animal, variations can hence occur in both the cell types and their number in these slices. Additionally, prior studies have shown that only a fraction of glial cells respond to bacterial insult with upregulation/activation of receptors (29). Given these constraints, dose response can therefore vary, as tissue slices are not identical. Hence, to be considered as effective in downregulating neuroinflammatory mediators for a given tissue, data was assessed cumulatively, with the following criteria. That the inhibitor significantly downregulates a given chemokine/cytokine for at least two of the three doses tested, and that this effect is seen for at least two of the three mediators tested.

With these criteria, the results were as follows: In the FC, in the presence of sonicated remnants, PD166866 significantly downregulated CCL2, CXCL8 and IL-6 from one tissue (#2), while CCL2 and IL-6 were significantly downregulated in the other (#5). In the presence of live bacteria, the inhibitor significantly downregulated CCL2 and CXCL8 in tissue #3, while CCL2 and IL-6 were significantly downregulated in the other FC tissue explant (#5) (**Table 2**).

In the DRG, the same evaluation criteria were used, and a similar result was seen in that at least two mediators were significantly downregulated. In the presence of non-viable remnants, CXCL8 and IL-6 were significantly downregulated in both DRG tissues (#5,6), while with live bacteria, they were CXCL8 and IL-6 in one (#5), and CCL2 and IL-6 in the other (#6). Thus, FGFR1 inhibitor was very effective in significantly downregulating at least two inflammatory mediators in both the FC and the DRG, and in all the tissues tested (**Table 2**).

### FGFR1-3 inhibitor is more effective in downregulating live *B. burgdorferi* mediated neuroinflammation than its remnants

The effect of FGFR1-3 inhibitor AZD4547 on inflammatory mediator release from FC and DRG tissues in response to either live or non-viable *B. burgdorferi* was similarly assessed, and is shown in **Table 3** (and **Supplementary Figures 6, 7**). Like the FGFR1 inhibitor, AZD4547 was also effective in downregulating inflammatory mediator levels induced by live *B. burgdorferi* in both FC and DRG. When using the same criteria for evaluation as for PD166866, at least two inflammatory mediators were significantly downregulated in all the FC and DRG tissues exposed to live bacteria and AZD4547. With non-viable *B. burgdorferi* remnants however, this effectiveness was somewhat diminished in that only one out of two tissues in the FC and two out of three tissues in the DRG showed a significant downregulation in chemokine/cytokine levels in response to FGFR1-3 inhibition. Tissue slices obtained from animal #5 in the FC, animal #9 in the DRG, failed to show a significant downregulation of most inflammatory markers in the presence of sonicated remnants and AZD4547 (**Table 3**).

**Table 3:** Fold^a^ change in chemokines/cytokines and apoptosis in response to AZD4547 FGFR1-3 inhibitor.

Tissue	Bacteria type	Animal #	Chemokines/Inhibitors	CCL2	CXCL8	IL-6	Apoptosis
**FC**	**Sonicated**	#**5**	3.3 µM AZD4547	**1.39**	***0.85***	1.08	0.98
			1.65 µM AZD4547	0.94	***0.69***	***0.72***	1.18
			330 nM AZD4547	***0.17***	**1.33**	***0.38***	1.26
			
		#**7**	3.3 µM AZD4547	**1.88**	1.09	**1.56**	**1.65**
			1.65 µM AZD4547	**1.52**	***0.72***	**1.65**	**1.67**
			330 nM AZD4547	**4.66**	***0.67***	**4.90**	**1.57**
			
	**Live**	#**5**	3.3 µM AZD4547	**14.32**	**2.71**	**5.79**	**1.43**
			1.65 µM AZD4547	**30.73**	**3.99**	**12.85**	1.38
			330 nM AZD4547	**10.70**	**3.74**	**5.06**	**2.01**
			
		#**8**	10 µM AZD4547	**1.84**	**1.38**	**2.80**	**1.86**
			1 µM AZD4547	1.47	**1.54**	**1.48**	**2.46**
			
**DRG**	**Sonicated**	#**9**	3.3 µM AZD4547	0.95	***0.64***	**1.90**	1.02
			1.65 µM AZD4547	0.93	***0.79***	**1.91**	1.00
			330 nM AZD4547	0.90	**1.83**	1.01	1.19
			
		#**10**	3.3 µM AZD4547	***0.73***	**2.46**	**7.37**	**1.40**
			330 nM AZD4547	1.08	**1.87**	**5.46**	**2.15**
			
		#**11**	3.3 µM AZD4547	1.22	**1.55**	**2.64**	1.19
			1.65 µM AZD4547	1.32	**2.16**	**2.54**	**1.62**
			330 nM AZD4547	**1.74**	**1.83**	**1.43**	**1.65**
			
	**Live**	#**9**	3.3 µM AZD4547	**4.17**	1.06	**1.20**	***0.80***
			1.65 µM AZD4547	**2.19**	**1.19**	**1.25**	**1.21**
			330 nM AZD4547	**5.25**	**1.66**	0.97	**1.37**
			
		#**11**	3.3 µM AZD4547	***0.67***	***0.53***	1.14	1.21
			1.65 µM AZD4547	**7.00**	**5.87**	**6.01**	**1.87**
			330 nM AZD4547	**3.41**	**2.35**	**2.99**	**1.56**

a Fold change was calculated as described in **Table 2** footnote, and significance as described therein. Calculated from **Supplementary Figures 6, 7** and **9**.

The overall effect of FGFR inhibitors in response to either treatment in FC or DRG tissues is shown in **Table 4**. Cumulatively, FGFR inhibition (both PD and AZD) downregulated CCL2 and IL-6 in 6/8 tissues, and CXCL8 in 4/8 tissues in the FC. In the DRG, 3/9, 7/9 and 9/9 tissues showed significant downregulation of CCL2, CXCL8 and IL-6 respectively (**Table 4**). Thus, in the FC, CCL2 and IL-6 were the most downregulated in response to FGFR inhibition, while in DRG they were CXCL8 and IL-6.

**Table 4:** The most significantly suppressed ^a^ inflammatory mediators in response to FGFR inhibition, and the overall efficacy of each FGFR inhibitor for the different bacterial treatments.

Tissue	Bacteria type	Treatment	CCL2	CXCL8	IL-6	Total
**FC**	**Sonicated**	PD166866	2/2	1/2	2/2	**5/6**
		AZD4547	1/2	0/2	1/2	**2/6**
	**Live**	PD166866	2/2	1/2	1/2	**4/6**
		AZD4547	1/2	2/2	2/2	**5/6**
		**Total**	**6/8**	**4/8**	**6/8**	
**DRG**	**Sonicated**	PD166866	0/2	2/2	2/2	**4/6**
		AZD4547	0/3	2/3	3/3	**5/9**
	**Live**	PD166866	1/2	1/2	2/2	**4/6**
		AZD4547	2/2	2/2	2/2	**6/6**
		**Total**	**3/9**	**7/9**	**9/9**	

a Assessment of the ability of FGFR inhibitors to suppress individual cytokine or chemokine was considered cumulatively within each experiment, over all the three doses. That is, for each inflammatory mediator, if two or more inhibitor doses significantly downregulated their expression, it was considered effective in downregulating that mediator (in that experiment). The numbers in the denominator indicate the total number of such assessments made. The numbers in the numerator indicate the number of assessments showing significant downregulation. Obtained from **Tables 2**, **3**. The grand totals are in bold.

Also, FGFR1 inhibitor PD166866 was better overall in suppressing inflammatory mediators mediated by either form of *B. burgdorferi* in both the FC and DRG. AZD4547, on the other hand, was much more effective with live bacteria mediated inflammatory mediator levels, compared to those mediated by *B. burgdorferi* remnants (**Table 4**).

### Inhibition of apoptosis reflects inflammatory inhibition

**Figure 5:**
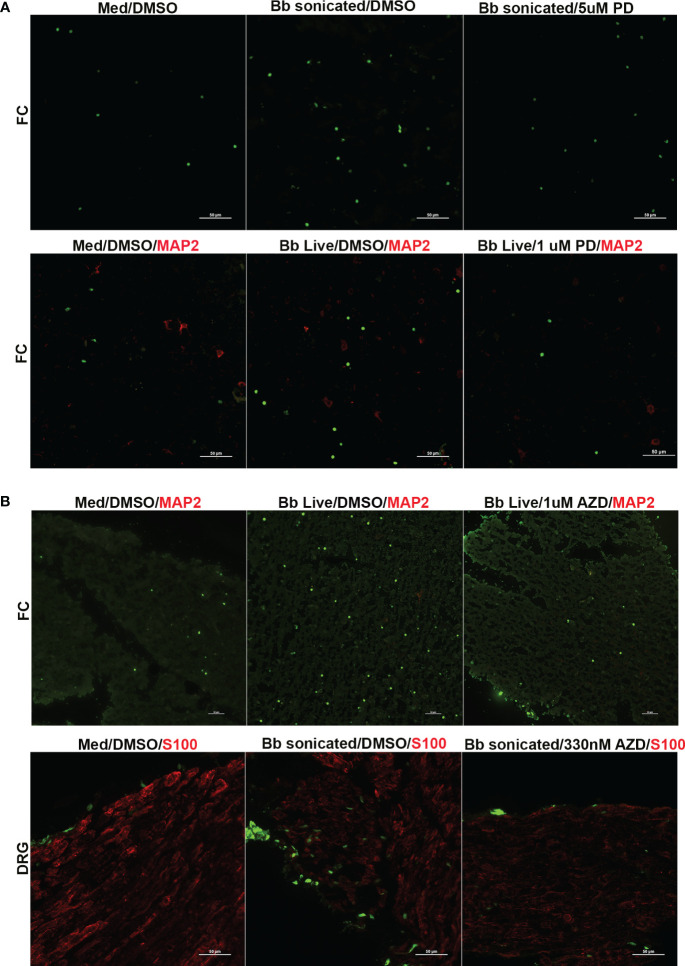
Immunofluorescence images of apoptosis in the FC and DRG tissues in response to FGFR inhibitors. Tissues exposed to live or sonicated *B burgdorferi* in the presence of **(A)** PD166866 (PD) or **(B)** AZD4547 (AZD) at various concentrations were analyzed for apoptosis levels by TUNEL assay and subjected to immunofluorescence analysis. Apoptotic nuclei are in green, while cells markers (if used) are in red (MAP2- neurons; S100- glia). In **(A)**, the top panel is from Animal 2, while the bottom panel is from Animal 3. In **(B)** top panel is from Animal 8, while the bottom panel is from Animal 10.

In our previous study we showed that both live and non-viable *B. burgdorferi* induced inflammatory mediators and apoptosis in *ex vivo* explants (28). Since FGFR inhibitors downregulated inflammatory mediator levels, their effect on apoptosis levels was then investigated. Results showed that overall, in those tissues where inflammatory mediator levels were effectively suppressed, apoptosis levels were also significantly diminished (**Tables 2**, **3**, **Supplementary Figures 8**, **9** and **Figure 5**). More specifically, for each dose experiment, where at least 2 of the 3 mediators were significantly downregulated, there was a corresponding significant downregulation of apoptosis levels (**Tables 2**, **3**, reading horizontally). This was true for majority of the doses that were tested for apoptosis. There were a few exceptions (6/47) where in two or more mediators were significantly suppressed, but the apoptosis was not (4 of the 6 exceptions), or only one mediator was significantly suppressed but it also had a significant downregulatory effect on apoptosis (2 of the 6). However, as mentioned before these were the exceptions rather than the rule. This also indicated that suppression of at least two mediators was needed to significantly downregulate apoptosis, and that FGFR inhibitors can effectively curb both neuroinflammatory levels as well as apoptotic levels.

Regarding the optimal dosage, low levels of PD166866 (1µM in FC and 100nM in DRG) and AZD45457 (330nM) were sufficient to downregulate both inflammatory levels and apoptosis in many of the tissues tested. While higher concentrations were also effective in suppressing cytokine/chemokine levels, there was a small uptick in apoptosis levels indicating possible mild toxicity. As FGFR inhibitors are clinically used at likely high doses to cause death of cancer cells, this is not surprising. However, this data also shows that at low doses, these inhibitors can achieve the opposite effect and be an effective anti-inflammatory therapeutic.

## Discussion

The study shows that FGFRs mediate an inflammatory response upon exposure to Lyme disease bacterium (or its remnants) in nervous tissues *ex vivo*, and that FGFR inhibitors can effectively curb the neuropathogenicity mediated by live or dead *B. burgdorferi*. The results are similar to our previous *in vitro* study on rhesus primary microglia (29), wherein rhesus microglia sourced from multiple animal tissues were shown to significantly upregulate FGFR1, 2 and 3 in response to live *B. burgdorferi*. In the current study, while this pattern of upregulation was evident in FC tissues, especially with FGFR1 and FGFR3 (**Figure 1**), it wasn’t always statistically significant. This could be due to the higher background staining in tissues or activation of cells in subsurface layers making enumeration somewhat difficult, compared to the cleaner *in vitro* single cell cultures. The expression patterns also varied among tissues from different animals (FGFR1 especially), indicating that genetic factors also influence FGFR expression in response to stimulus. (However, it is also possible that the antibodies used in immunofluorescence do not recognize all the splice variants of FGFRs). Compared to single cell cultures of microglia, FGFR2 and FGFR3 expression in the FC tissues were higher in explants containing just medium. This indicated that cells other than microglia in the FC express these receptors under normal conditions that may be modulated upon stimulation. FGFR1 and FGFR3 were the most modulated and significantly upregulated with bacterial treatments (**Figure 1**). The results are similar to those seen in neurological diseases such as Alzheimer’s disease (AD) and multiple sclerosis (MS) where increased FGFR1 staining in white matter of AD and MS patients has been reported (36, 37). Similarly, increased FGFR1 expression in the prefrontal cortex of patients with major depressive disorder has also been demonstrated (38). Increased FGFR3 staining has been documented in the anterior cingulate cortex of patients with Lewy Body dementia (LBD) (39). Overexpression of FGFR3 in a gain-of-function mutation in hippocampus of mice has been shown to induce memory deficits (40). Interestingly, CNS manifestations of Lyme neuroborreliosis include cognitive impairment, depression, obsessive compulsive disorders, schizophrenia, and dementia-like syndromes (41). The presence of Lyme disease bacterium has also been demonstrated in the brains of patients with AD and LBD (42, 43). Upregulation of FGFRs in response to Lyme disease bacterium in the brain tissues and a similar response in these neurological conditions reiterate the idea of mechanistic commonalities contributing to these phenotypes in Lyme neuroborreliosis (29).

The individual cell types expressing specific FGFRs in response to live/remnant treatment were also explored. As microglial and oligodendrocyte FGFR expression patterns have been largely analyzed in our previous studies (29, 31), determination of cell specificity was confined to astrocytes and neurons. The data showed that FGFR1 was primarily expressed in astrocytes, while the other two were predominantly expressed in neurons- although all three-receptor staining could be seen in both cells in response to treatments (**Figures 3**, **4**). In rodent brains, FGFR1 was shown to have a neuronal predominance while FGFR2 and FGFR3 had a glial preference (44–46) contrary to these results. The differences could be due to many reasons, including species differences (rodents vs rhesus), method of identification (mRNA vs protein), treatments, or region differences (diverse brain regions vs frontal cortex). Additionally, not all cell types were tested in these studies, making exact comparisons difficult. In human hippocampus FGFR1 is expressed in neurons and FGFR3 in astrocytes (47). However, in human cortex (both normal and Alzheimer’s), FGFR1 was shown to be expressed in astrocytes like this data (37). In the LBD study, higher FGFR3 expression in the anterior cingulate cortex was in neurons (39), similar to the neuronal specificity in this study, indicating potential translatability to humans.

In the DRG, FGFR expression was present in both medium exposed explants as well as in the treated sections, with differences not discernible by immunofluorescence (**Supplementary Figure 3**). Lower dilutions of the antibodies did not yield a visible signal. Determination of the phosphorylated state of FGFR1 (Tyr 653, 654) showed high background in both control sections and sections exposed to bacteria/remnants (not shown). Since the DRG sections did respond to two different FGFR inhibitors, (**Tables 2**, **3**), it is likely that they are activated, but perhaps at a different tyrosine residue. The differences, if there are any, remain to be explored by protein quantitation or by analysis of phosphorylation at a different tyrosine residue (48). It is also possible that differences as discerned by immunohistochemistry manifest over a longer time. In the DRG, upregulation of phosphorylated FGFR1 was seen 3 days after nerve injury in a rat model of neuropathic pain, while no changes were seen in the mRNA levels (49). As the tissues used in our *ex vivo* study are short-lived, long-lasting organ cultures may be needed to study such effects. Cell specificity of these receptors showed that they were expressed in both neurons and (satellite) glial cells, in both medium and *B. burgdorferi* exposed conditions (**Supplementary Figure 3**). Individual cell cultures are needed to discern cell specific differences in expression and over time, as tissue sections showed variations in cell numbers/staining precluding uniform enumerations.

As FGFRs were shown to mediate inflammatory mediator production in response to Lyme disease bacteria in microglia (29), specific FGFR inhibitors were used to test their efficacy in downregulating inflammatory mediators in nervous tissues. The results are shown in **Tables 2**–**4** (and **Supplementary Figures 4–9**). The two inhibitors tested (FGFR1 inhibitor and FGFR1-3 inhibitor) were efficacious in downregulating inflammatory mediator production in response to live bacteria in both FC and DRG. In response to non-viable remnants however, while FGFR1 inhibitor (PD1666866) was efficacious in downregulating inflammatory response in both FC and DRG, AZD4547, the FGFR1-3 inhibitor, was less so. A significant downregulatory effect on cytokines in the presence of AZD45457 and non-viable remnants was seen in only one of the two FC tissues or 2 of the three DRG tissues tested. Interestingly, this opposite effect (AZD being efficacious with live but not with non-viable) was seen in the tissues derived from the same animal (#5 in the FC and #9 in the DRG; **Table 3**), indicating that this was not due to genetic variability. It is possible that variations in tissue slices could contribute to this effect. It is also possible that non-viable fragments activate several additional proinflammatory pathways in those tissues whose effect superseded those mediated by FGFRs. Additionally, while the individual FGFRs were shown to mediate inflammatory pathways in primary microglia in response to *B. burgdorferi*, their role in other cells such as astrocytes and neurons is not known. So, it is possible that in some of these cells or in some tissues, they (particularly FGFR2 or FGFR3) have anti-inflammatory effects that interfere with the pro-inflammatory effects mediated by FGFR1. Depending on the strength of that interference, the efficacy of AZD treatment would vary. This hypothesis remains to be tested. However, since AZD4547 was efficacious 60% (3/5; **Table 3**) of the time in downregulating neuroinflammatory mediators in response to non-viable *B. burgdorferi*, it still had a significant anti-inflammatory effect.

In a previous study, the inflammatory mediator production as induced by non-viable fragments was shown to be significantly higher than those induced by live *B. burgdorferi*, particularly in the FC (28). In the current study, these large differences were not seen when DMSO (solvent control) was added to live/non-viable or medium controls. DMSO is a modulator of inflammation, largely demonstrated to be anti-inflammatory (50). Hence it is not surprising that the large differences seen in the prior study without the addition of DMSO was significantly reduced in its presence. So, it is conceivable that DMSO itself could be a treatment option for some patients. However, it has so far been largely used for topical applications as limited safety data is available for long term ingestion (51, 52). Also, significant amounts of inflammatory mediators can remain even when DMSO is present, as seen in **Supplementary Figures 4–7**, indicating that DMSO treatment alone may be an insufficient anti-inflammatory supplemental therapeutic for persistent neuroinflammation in Lyme patients.

Multiple studies have shown that production of innate immune mediators in neurological Lyme is accompanied by apoptosis in response to live and non-viable *B. burgdorferi*, be it *in vitro*, *ex vivo* or *in vivo* studies (27, 28, 30, 53–56). In concurrence with these studies, increased apoptosis was seen in tissues in response to either live or non-viable fragments that also significantly upregulated inflammatory mediators. Accordingly, suppression of inflammatory mediators by the FGFR inhibitors also downregulated apoptosis levels and failed to do so if there was no effect on the former. This effect was seen majority of the time in all the tissues tested. There were a few exceptions (6/47), however, where in significant suppression of two mediators for a given dose did not elicit a significant downregulatory effect on apoptosis, or vice versa, where in cytokines were not downregulated significantly but apoptosis was significantly downregulated. The reasons are likely manifold. 1) High dose of inhibitor having a toxic effect, where cytokines are downregulated but had adverse effects on viability (1/6); 2) significant suppression of mediator levels also downregulated apoptosis, just not significantly so (3/6), and 3) inhibitors likely downregulating other mediators not tested (2/6). Overall, the study showed that FGFRs are attractive targets for anti-inflammatory therapeutics for neurological Lyme disease. FGFRs have been targeted in other neurological conditions as well. Deletion of *Fgfr1* or *Fgfr2* in oligodendrocytes was shown to inhibit myelin and axon degeneration in mouse models of experimental autoimmune encephalitis (a multiple sclerosis model) (57, 58). Inhibition of FGFR1 with an FGFR inhibitor has been demonstrated to reduce neuropathic pain in a rat model of peripheral nerve injury (49). *Fgfr3* was shown to mediate neuronal cell death in the ganglia (59) in a mouse model of nerve injury. *Fgfr3* knock out mice had normal number of spinal ganglionic neurons compared to the wild type indicating that this receptor is involved in neuronal cell death in the PNS.

Of the two FGFR inhibitors used in this study, one is an orally bioavailable drug (AZD4547) while the other is used for research purposes. While AZD4547 has been tested clinically for a patient with astrocytoma, it is not clear if it can effectively cross the blood-brain barrier, especially at low doses. Additionally, adverse events reported with this drug in patients (60) indicates that toxicity might be an issue, although tolerability has been reported elsewhere (61).

However, this study also showed that chemokines and cytokines could be potential direct targets for therapeutics. CCL2/IL-6 and CXCL8/IL-6 were the most downregulated chemokines/cytokines in response to FGFR inhibition in the CNS and PNS respectively. Inhibition of a single chemokine/cytokine did not affect apoptosis in general, but suppression of two of the three mediators were sufficient to downregulate apoptosis in most cases (**Tables 2**–**4**). Therefore, double biologics that target these mediators may be alternatively used (in lieu of FGFR inhibition) as supplemental therapeutics for persistent neuroinflammation.

In summary, the data presented in this study show the role of FGFRs in neurological Lyme disease in nervous system tissues reinforcing the results of our previous *in vitro* study in primary microglia (29). The study also demonstrates proof-of-principle for the effectiveness of FGFR inhibitors as anti-inflammatory therapeutics in neurological Lyme, and additionally identifies specific chemokine/cytokines as potential targets for biologics to alleviate neuroinflammatory conditions in Lyme disease.

## Data availability statement

The original contributions presented in the study are included in the article/**Supplementary Material**. Further inquiries can be directed to the corresponding author.

## Ethics statement

According to Tulane IACUC policy, tissues obtained after an animal has been euthanized and was not euthanized for the purpose of that study are not subject to IACUC approval. The studies were conducted in accordance with the local legislation and institutional requirements.

## Author contributions

GP: Conceptualization, Data curation, Formal analysis, Funding acquisition, Investigation, Methodology, Validation, Writing – original draft, Writing – review & editing.

## Supplementary material

Supplementary Figure 1 Negative control for immunohistochemistry. The figure shows the lack of immunofluorescence in an FC tissue probed only with the secondary antibody conjugated to Alexa 488, and no primary antibody. Animal #2 tissue (exposed to Medium) is shown at different areas and at different magnification. DAPI stained nuclei in blue. Related to **Figure 2**.

Supplementary Figure 2 MAP2 staining of neurons in Frontal cortex. FC tissues from different animals (exposed to Live *B. burgdorferi*) were stained for MAP2(red), FGFR3 (green) and DAPI (blue, nuclear stain). Figure shows that not all neurons stained uniformly for MAP2 but could be identified as likely neurons by the large nucleus that stains less intensely with DAPI. Arrows indicate neuronal cells based on such criteria. In animal #2 tissue, yellow colocalization of the red MAP2 and green FGFR3 can be seen in the inset panel. Related to **Figure 4** (and **Figure 3**).

Supplementary Figure 3 FGFR 1-3 expression from rhesus dorsal root ganglion (DRG) in response to live and non-viable *B. burgdorferi*. DRG tissues exposed to live or nonviable *B. burgdorferi* were analyzed for FGFR1-3 (green) expressions. Expression from medium only tissues is also shown. FGFR1 and FGFR3 expressions are from Animal 3, while FGFR2 is from Animal 4. Bar represents 50 µm unless indicated. Blue pseudo color is shown for neuronal areas in FGFR1/FGFR3 panels. In the FGFR2 panels, the blue color indicates DAPI stain, while NeuN (neuronal nuclei) staining for neurons is in red.

Supplementary Figure 4 Effect of PD166866 on inflammatory mediator production from FC tissues in response to sonicated or live *B. burgdorferi*. Supernatants collected from the *ex vivo* assays in response to PD166866 treatments were analyzed for CCL2, CXCL8 and IL-6. **(A)** FC tissues and sonicated bacteria, **(B)** FC tissues and Live bacteria. Bb/S-sonicated *B. burgdorferi*, Bb/L- Live *B. burgdorferi*, Med-Medium. D-DMSO, PD-PD166866. All comparisons are with Bb/DMSO group. *p< 0.05; **p< 0.01, *** p< 0.001, **** p < 0. 0001; ns- not significant. Black lines show treatment doses that significantly lower inflammatory mediators, while red lines show a significant increase in inflammatory output compared to Bb/DMSO treatment alone. Medium/DMSO served as negative control for inflammatory mediator induction. Animal numbers are indicated on the graphs. Related to **Table 2**.

Supplementary Figure 5 Effect of PD166866 on inflammatory mediator production from DRG tissues in response to sonicated or live *B. burgdorferi*. DRG tissue slices were exposed to sonicated or live *B. burgdorferi* in the presence of PD166866 for 4 hours, and the indicated inflammatory mediators were analyzed in the supernatants by a multiplex assay. **(A)** DRG tissues and sonicated bacteria, **(B)** DRG tissues and Live bacteria. Bb/S-sonicated *B. burgdorferi*, Bb/L- Live *B. burgdorferi*, Med-Medium. D-DMSO, PD-PD166866. All comparisons are with Bb/DMSO group. *p< 0.05; **p< 0.01, *** p< 0.001, **** p < 0. 0001; ns- not significant. As indicated in supplementary figure 4 legend, black lines and red lines show significant downregulation and upregulation respectively, compared to Bb/DMSO treatment alone. Medium/DMSO served as negative control for inflammatory mediator induction. Animal tissues sourced for each experiment are indicated on each graph. Related to **Table 2**.

Supplementary Figure 6 Effect of AZD4547 on inflammatory mediator production from FC tissues in response to sonicated or live *B. burgdorferi*. CCL2, CXCL8 and IL-6 levels in the supernatants collected from *ex vivo* tissue explants in response to AZD4547 treatments are shown. **(A)** FC tissues and sonicated bacteria, **(B)** FC tissues and Live bacteria. Bb/S-sonicated *B. burgdorferi*, Bb/L- Live *B. burgdorferi*, Med-Medium. D-DMSO, AZD-AZD4547. Like Supplemental figures 4 and 5, all comparisons are with Bb/DMSO group. *p< 0.05; **p< 0.01, *** p< 0.001, **** p < 0. 0001. Again, black lines show treatment doses that significantly lower inflammatory mediators, while red lines show a significant increase in inflammatory output compared to Bb/DMSO treatment alone. Ns indicates not significant. Animal numbers are indicated after the hashtag. Related to **Table 3**.

Supplementary Figure 7 Effect of AZD4547 on inflammatory mediator production from DRG tissues in response to sonicated or live *B. burgdorferi*. **(A)** shows inflammatory mediator output from DRG tissues in response to sonicated *B. burgdorferi* and various doses of AZD4547. **(B)** shows a similar analysis conducted with live *B. burgdorferi*. Bb/S-sonicated *B. burgdorferi*, Bb/L- Live *B. burgdorferi*, Med-Medium. D-DMSO, AZD-AZD4547. *p< 0.05; **p< 0.01, *** p< 0.001, **** p < 0. 0001; ns- not significant. All comparisons are with the Bb/DMSO group. Black and red lines signify significantly downregulated and upregulated mediators respectively, as indicated previously. Medium tissues with DMSO served as negative control for inflammatory mediator production. The animal tissues used for the experiment are indicated on the graph. Related to **Table 3**.

Supplementary Figure 8 Apoptosis levels in the FC and DRG tissues in response to inhibitor PD166866. *Ex vivo* explants subjected to various treatment combinations were fixed in paraformaldehyde, and cryo-preserved. Cryosections from various treatment groups were analyzed by TUNEL assay for apoptosis levels. A semi-quantitative analysis was performed by counting apoptotic nuclei under an immunofluorescent microscope, and the results were graphed. *p< 0.05; **p< 0.01, *** p< 0.001, **** p < 0. 0001; ns- not significant Bb/S-sonicated *B. burgdorferi*, Bb/L- Live *B. burgdorferi*, Med-Medium. D-DMSO, PD-PD166866. All comparisons are with Bb/DMSO group. Black lines show treatment doses that significantly lower apoptotic levels compared to Bb/DMSO treatment alone. Medium/DMSO treatment served as a negative control for apoptosis. Treatment combinations were as follows. **(A)** FC/ Sonicated bacteria. **(B)** FC/ Live bacteria. **(C)** DRG /Sonicated bacteria. **(D)** DRG /Live bacteria. Animal numbers for each experiment are indicated after the hashtag. Related to **Table 2**.

Supplementary Figure 9 Apoptosis levels in the FC and DRG tissues in response to inhibitor AZD4547. Apoptosis levels in response to AZD4547 treatment were measured by the TUNEL assay as described in the methods. **(A)** FC/ Sonicated bacteria. **(B)** FC/ Live bacteria. **(C)** DRG /Sonicated bacteria. **(D)** DRG /Live bacteria. Bb/S-sonicated *B. burgdorferi*, Bb/L- Live *B. burgdorferi*, Med-Medium. D-DMSO, AZD-AZD4547. *p< 0.05; **p< 0.01, *** p< 0.001, **** p < 0. 0001; ns- not significant. All comparisons are with Bb/DMSO group. Black lines and red lines show significantly lower and significantly higher apoptotic levels respectively, compared to Bb/DMSO treatment alone. Medium/DMSO treatment served as a negative control for apoptosis. Animal tissues sourced for each experiment are indicated on each graph. Related to **Table 3**.
