# Supplementary figures and images for: Fibroblast growth factor receptor inhibitors mitigate the neuropathogenicity of Borrelia burgdorferi or its remnants ex vivo
Source: Front Immunol. 2024 Apr 4;15:1327416. doi: 10.3389/fimmu.2024.1327416 (PMC11024320; doi:10.3389/fimmu.2024.1327416)

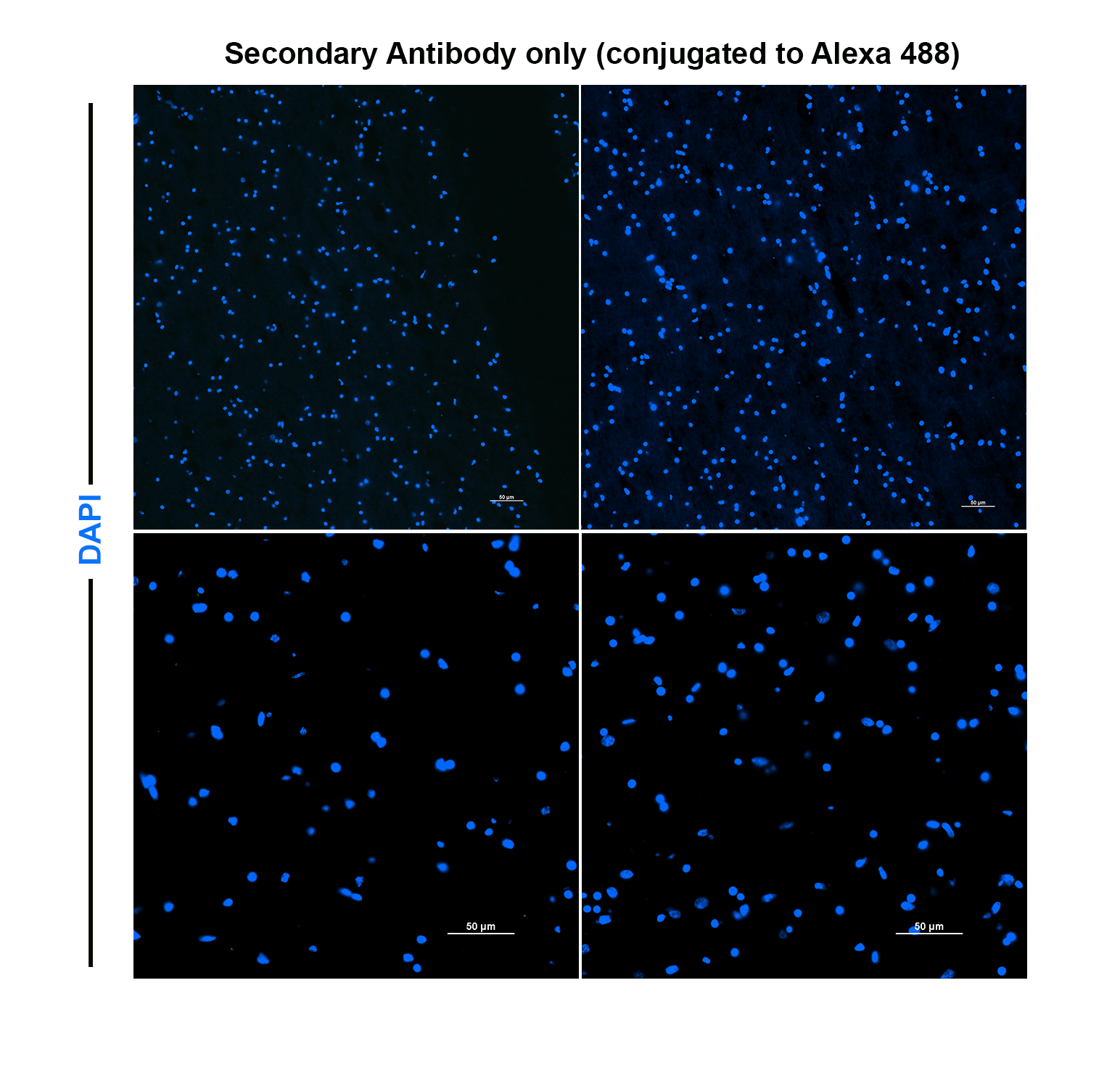

Supplement: Supplementary Figure 1 — Negative control for immunohistochemistry. The figure shows the lack of immunofluorescence in an FC tissue probed only with the secondary antibody conjugated to Alexa 488, and no primary antibody. Animal #2 tissue (exposed to Medium) is shown at different areas and at different magnification. DAPI stained nuclei in blue. Related to Figure 2 . [file Image_1.tif]

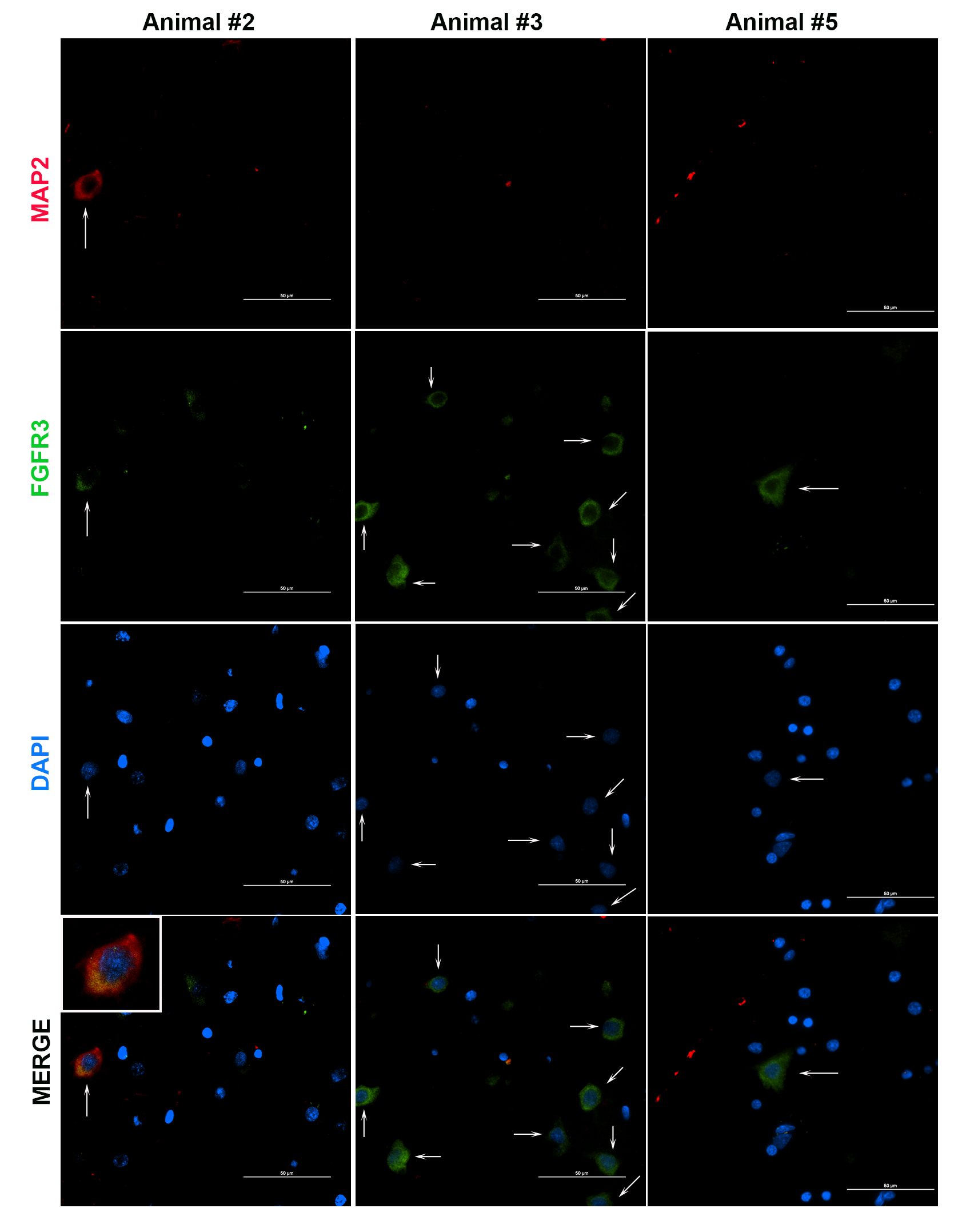

Supplement: Supplementary Figure 2 — MAP2 staining of neurons in Frontal cortex. FC tissues from different animals (exposed to Live B. burgdorferi) were stained for MAP2(red), FGFR3 (green) and DAPI (blue, nuclear stain). Figure shows that not all neurons stained uniformly for MAP2 but could be identified as likely neurons by the large nucleus that stains less intensely with DAPI. Arrows indicate neuronal cells based on such criteria. In animal #2 tissue, yellow colocalization of the red MAP2 and green FGFR3 can be seen in the inset panel. Related to Figure 4 (and Figure 3 ). [file Image_2.tif]

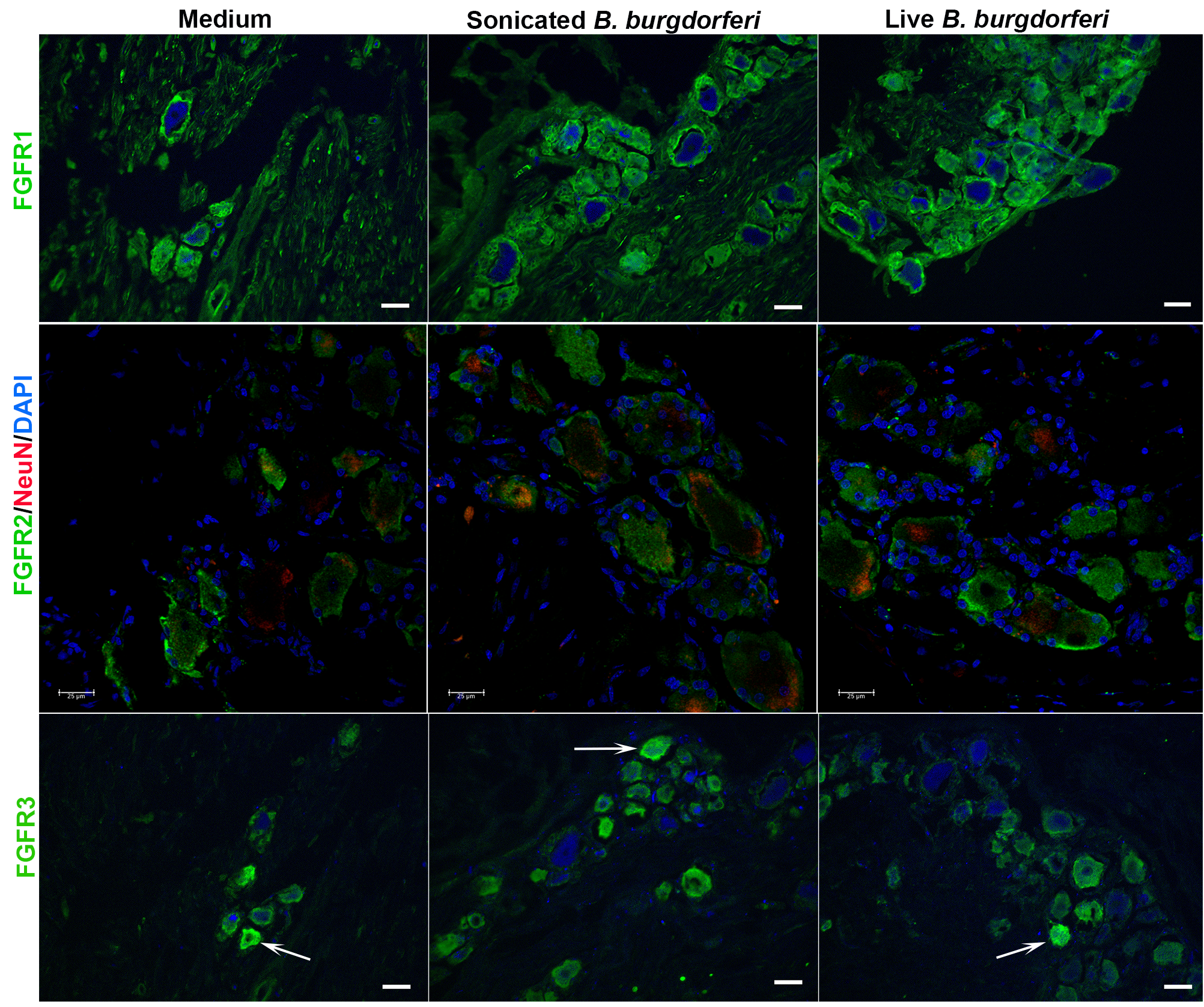

Supplement: Supplementary Figure 3 — FGFR 1-3 expression from rhesus dorsal root ganglion (DRG) in response to live and non-viable B. burgdorferi. DRG tissues exposed to live or nonviable B. burgdorferi were analyzed for FGFR1-3 (green) expressions. Expression from medium only tissues is also shown. FGFR1 and FGFR3 expressions are from Animal 3, while FGFR2 is from Animal 4. Bar represents 50 µm unless indicated. Blue pseudo color is shown for neuronal areas in FGFR1/FGFR3 panels. In the FGFR2 panels, the blue color indicates DAPI stain, while NeuN (neuronal nuclei) staining for neurons is in red. [file Image_3.tif]

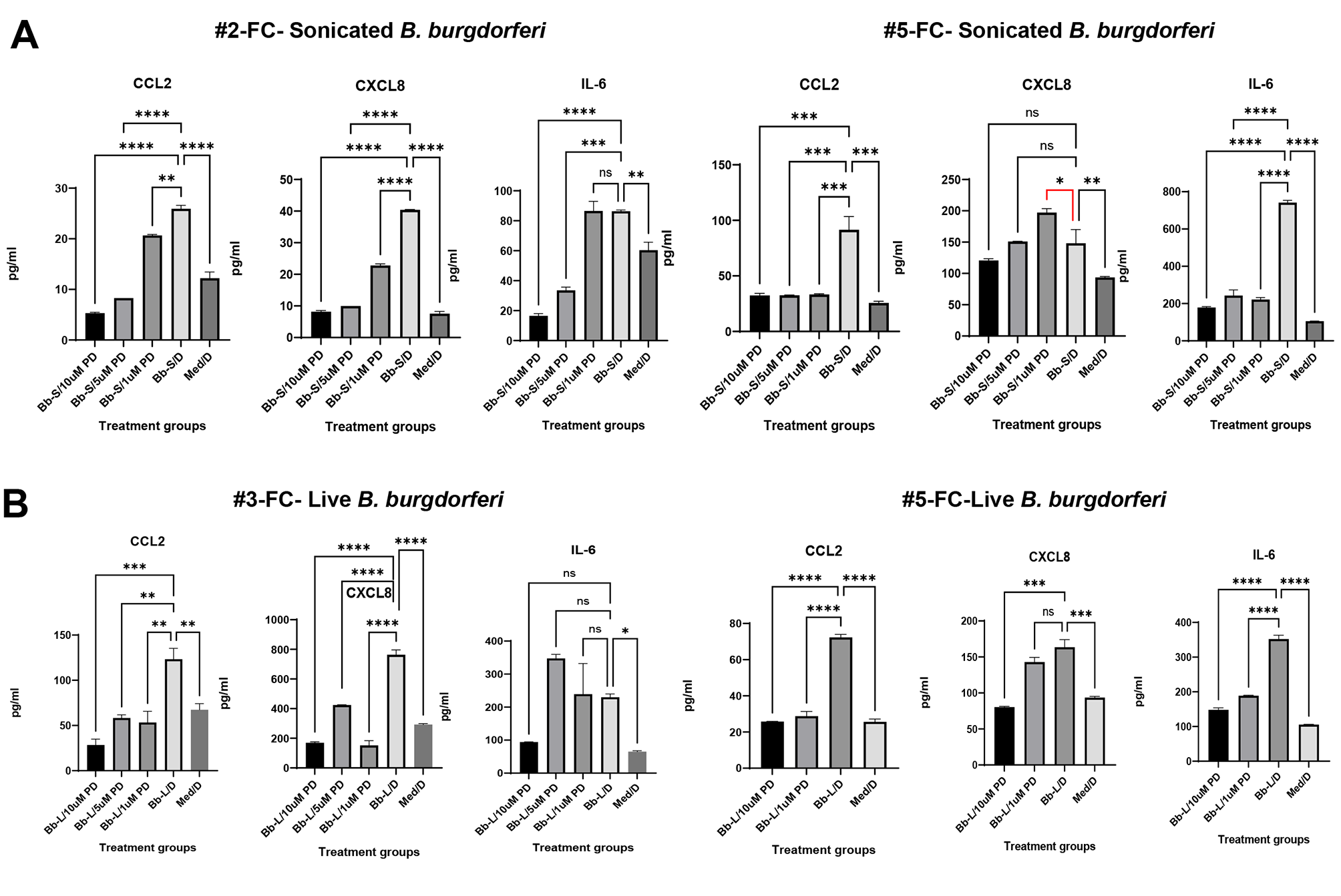

Supplement: Supplementary Figure 4 — Effect of PD166866 on inflammatory mediator production from FC tissues in response to sonicated or live B. burgdorferi. Supernatants collected from the ex vivo assays in response to PD166866 treatments were analyzed for CCL2, CXCL8 and IL-6. (A) FC tissues and sonicated bacteria, (B) FC tissues and Live bacteria. Bb/S-sonicated B. burgdorferi, Bb/L- Live B. burgdorferi, Med-Medium. D-DMSO, PD-PD166866. All comparisons are with Bb/DMSO group. *p< 0.05; **p< 0.01, *** p< 0.001, **** p < 0. 0001; ns- not significant. Black lines show treatment doses that significantly lower inflammatory mediators, while red lines show a significant increase in inflammatory output compared to Bb/DMSO treatment alone. Medium/DMSO served as negative control for inflammatory mediator induction. Animal numbers are indicated on the graphs. Related to Table 2 . [file Image_4.tif]

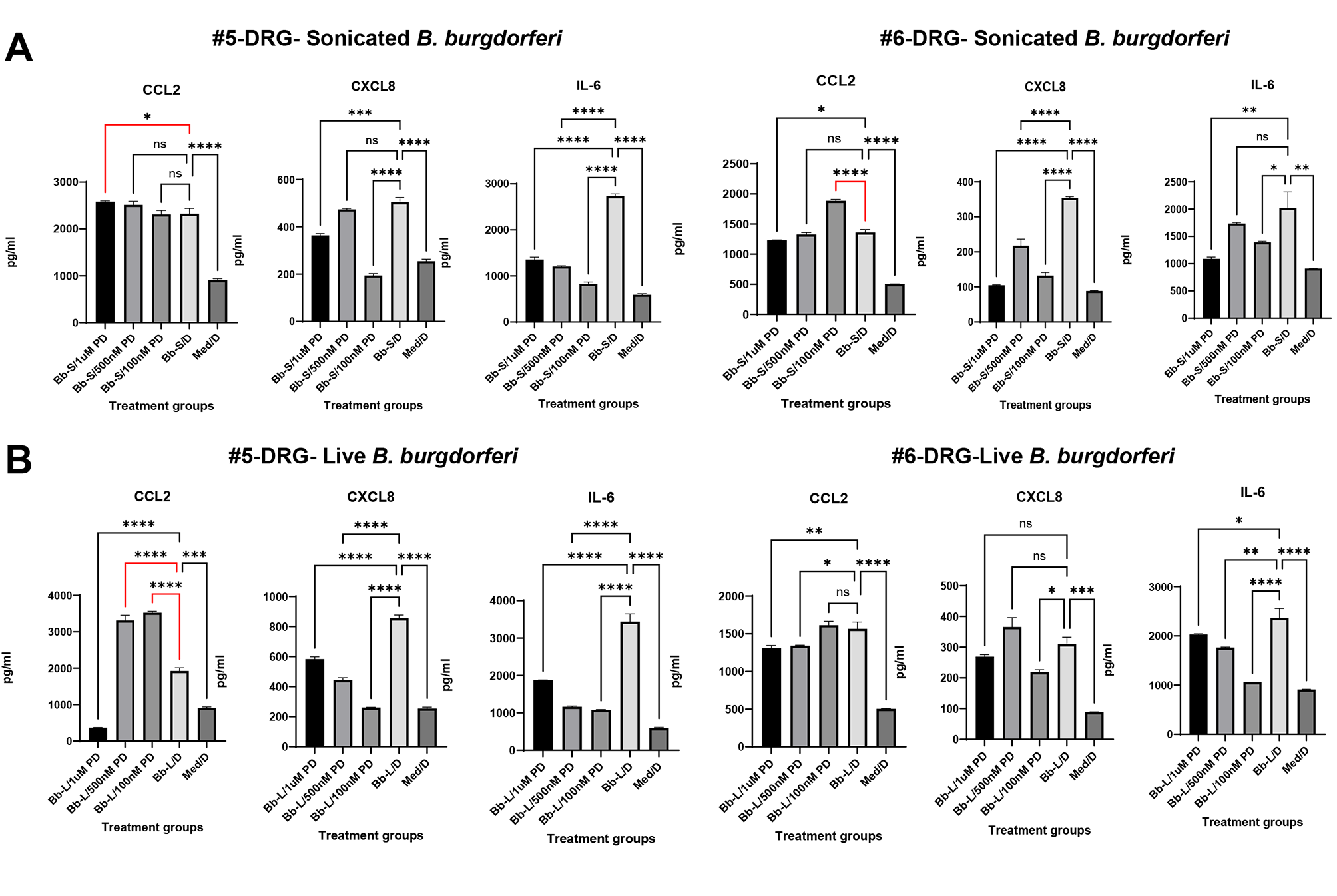

Supplement: Supplementary Figure 5 — Effect of PD166866 on inflammatory mediator production from DRG tissues in response to sonicated or live B. burgdorferi. DRG tissue slices were exposed to sonicated or live B. burgdorferi in the presence of PD166866 for 4 hours, and the indicated inflammatory mediators were analyzed in the supernatants by a multiplex assay. (A) DRG tissues and sonicated bacteria, (B) DRG tissues and Live bacteria. Bb/S-sonicated B. burgdorferi, Bb/L- Live B. burgdorferi, Med-Medium. D-DMSO, PD-PD166866. All comparisons are with Bb/DMSO group. *p< 0.05; **p< 0.01, *** p< 0.001, **** p < 0. 0001; ns- not significant. As indicated in supplementary figure 4 legend, black lines and red lines show significant downregulation and upregulation respectively, compared to Bb/DMSO treatment alone. Medium/DMSO served as negative control for inflammatory mediator induction. Animal tissues sourced for each experiment are indicated on each graph. Related to Table 2 . [file Image_5.tif]

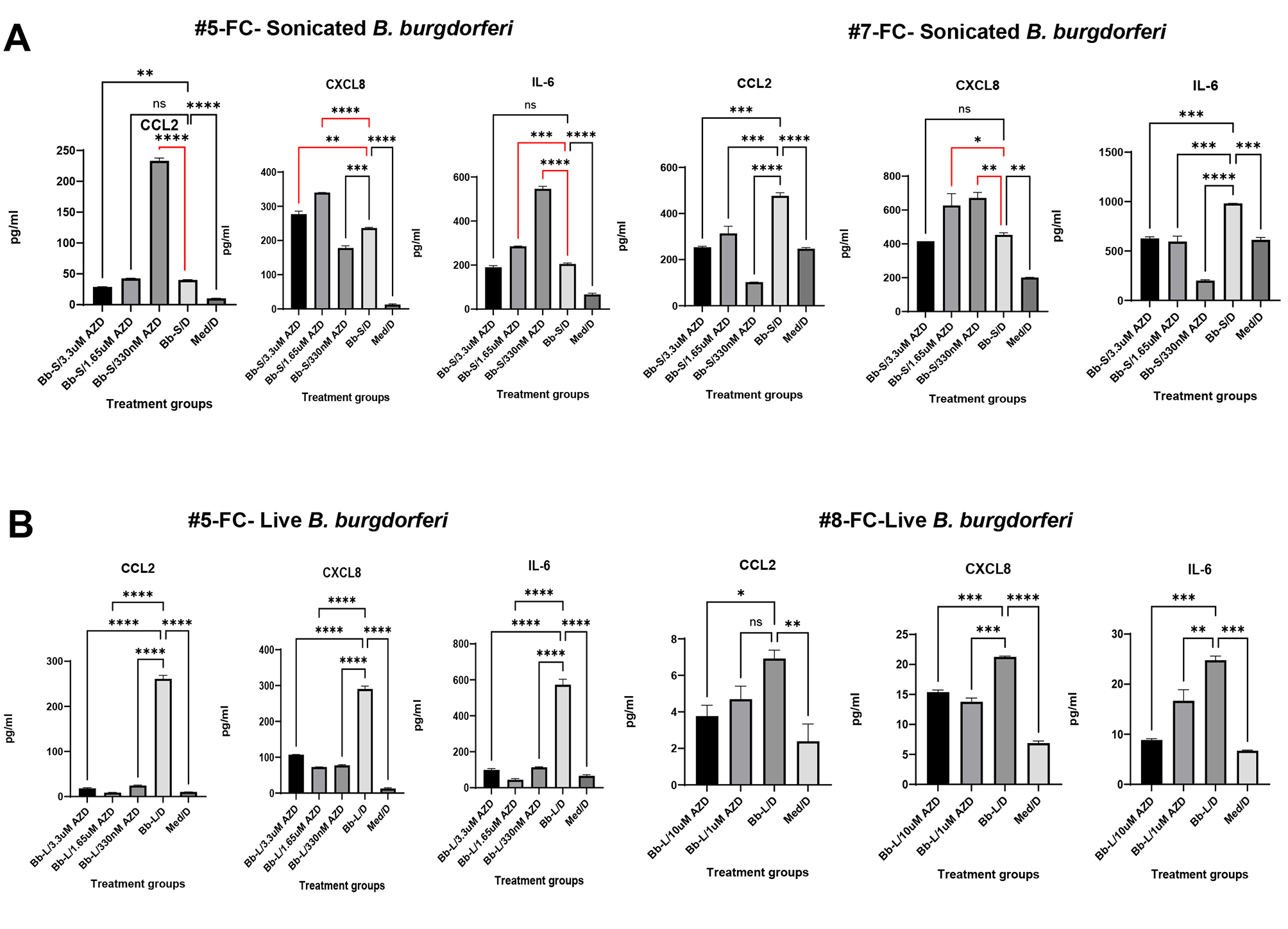

Supplement: Supplementary Figure 6 — Effect of AZD4547 on inflammatory mediator production from FC tissues in response to sonicated or live B. burgdorferi. CCL2, CXCL8 and IL-6 levels in the supernatants collected from ex vivo tissue explants in response to AZD4547 treatments are shown. (A) FC tissues and sonicated bacteria, (B) FC tissues and Live bacteria. Bb/S-sonicated B. burgdorferi, Bb/L- Live B. burgdorferi, Med-Medium. D-DMSO, AZD-AZD4547. Like Supplemental figures 4 and 5, all comparisons are with Bb/DMSO group. *p< 0.05; **p< 0.01, *** p< 0.001, **** p < 0. 0001. Again, black lines show treatment doses that significantly lower inflammatory mediators, while red lines show a significant increase in inflammatory output compared to Bb/DMSO treatment alone. Ns indicates not significant. Animal numbers are indicated after the hashtag. Related to Table 3 . [file Image_6.tif]

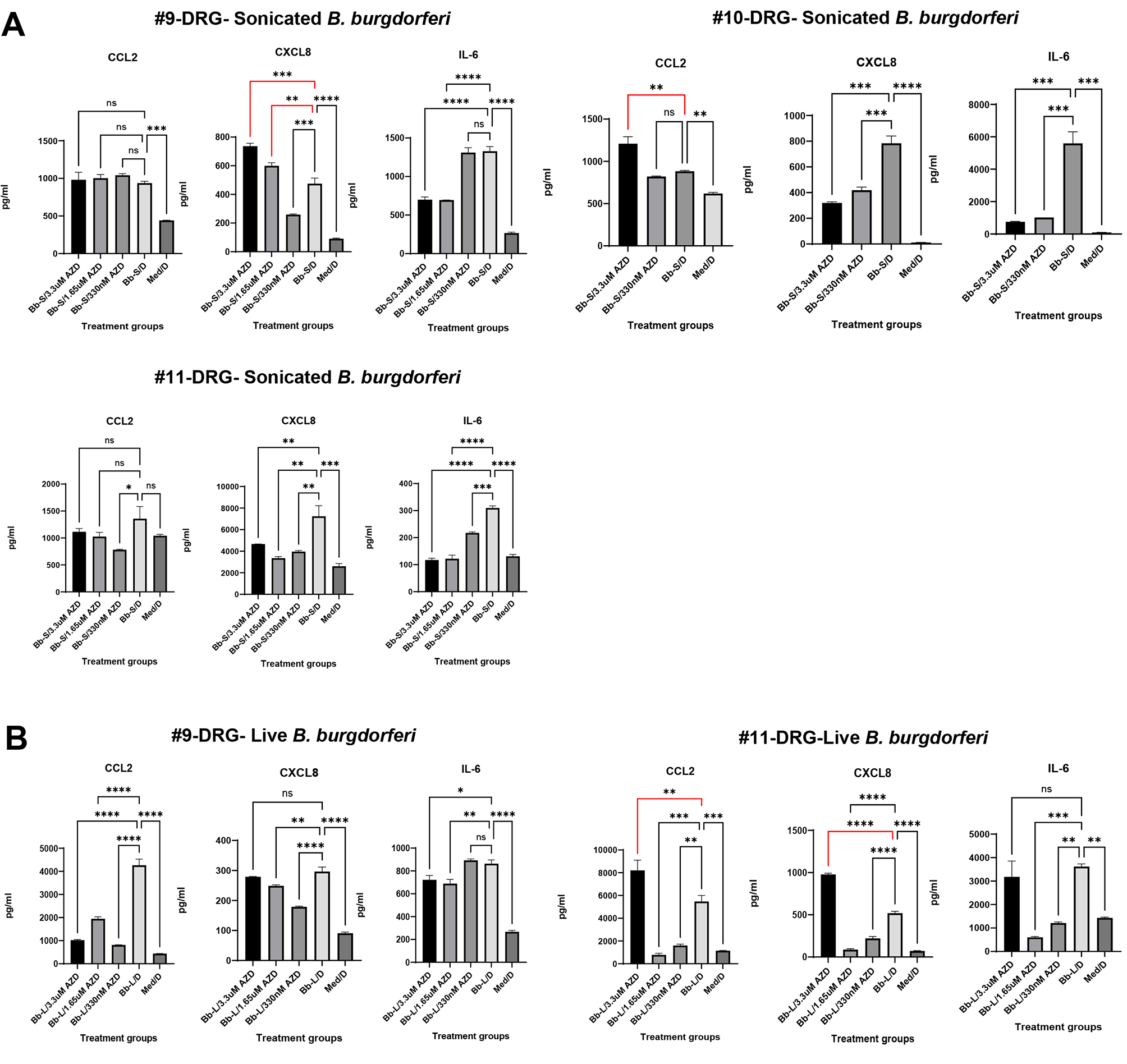

Supplement: Supplementary Figure 7 — Effect of AZD4547 on inflammatory mediator production from DRG tissues in response to sonicated or live B. burgdorferi. (A) shows inflammatory mediator output from DRG tissues in response to sonicated B. burgdorferi and various doses of AZD4547. (B) shows a similar analysis conducted with live B. burgdorferi. Bb/S-sonicated B. burgdorferi, Bb/L- Live B. burgdorferi, Med-Medium. D-DMSO, AZD-AZD4547. *p< 0.05; **p< 0.01, *** p< 0.001, **** p < 0. 0001; ns- not significant. All comparisons are with the Bb/DMSO group. Black and red lines signify significantly downregulated and upregulated mediators respectively, as indicated previously. Medium tissues with DMSO served as negative control for inflammatory mediator production. The animal tissues used for the experiment are indicated on the graph. Related to Table 3 . [file Image_7.tif]

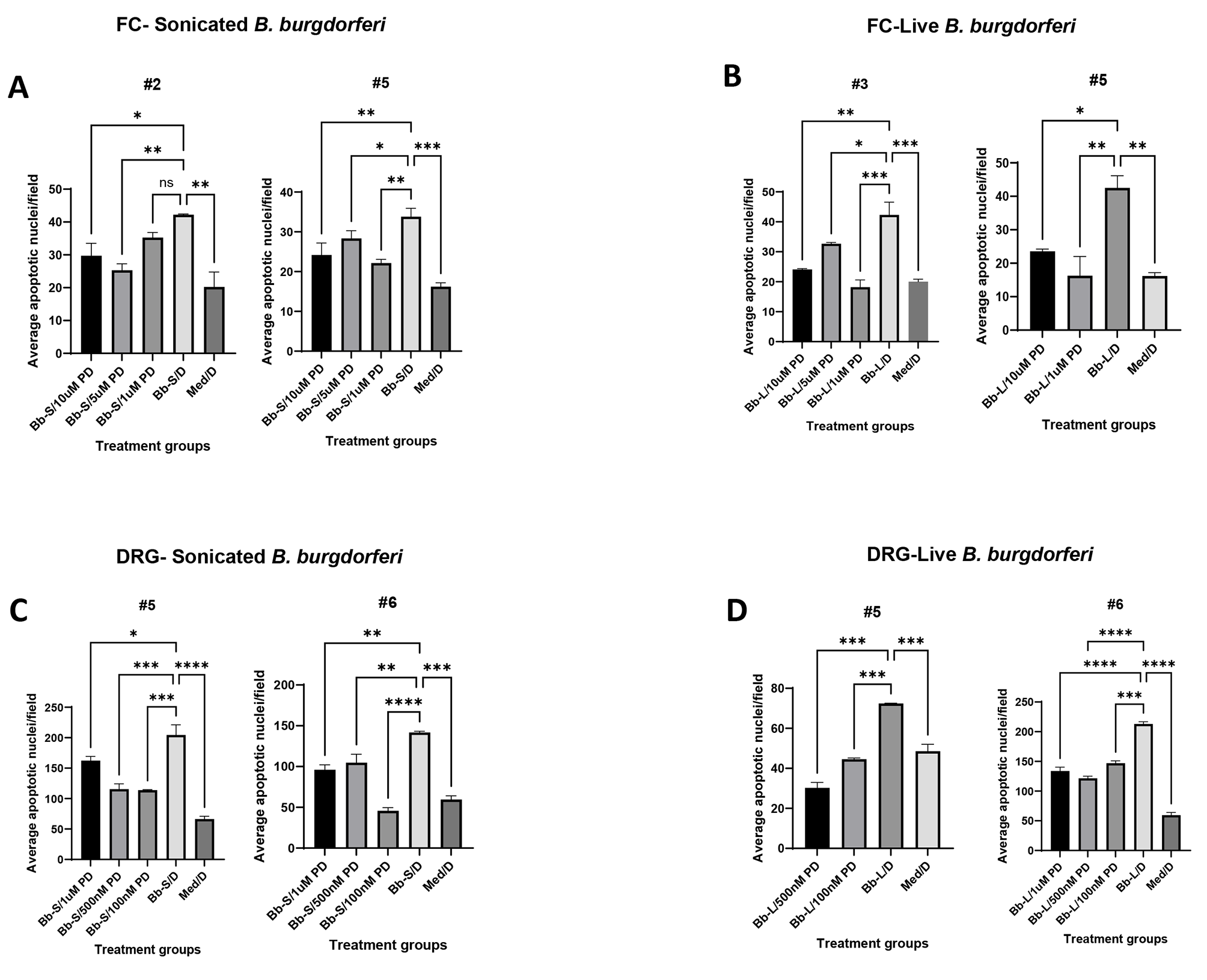

Supplement: Supplementary Figure 8 — Apoptosis levels in the FC and DRG tissues in response to inhibitor PD166866. Ex vivo explants subjected to various treatment combinations were fixed in paraformaldehyde, and cryo-preserved. Cryosections from various treatment groups were analyzed by TUNEL assay for apoptosis levels. A semi-quantitative analysis was performed by counting apoptotic nuclei under an immunofluorescent microscope, and the results were graphed. *p< 0.05; **p< 0.01, *** p< 0.001, **** p < 0. 0001; ns- not significant Bb/S-sonicated B. burgdorferi, Bb/L- Live B. burgdorferi, Med-Medium. D-DMSO, PD-PD166866. All comparisons are with Bb/DMSO group. Black lines show treatment doses that significantly lower apoptotic levels compared to Bb/DMSO treatment alone. Medium/DMSO treatment served as a negative control for apoptosis. Treatment combinations were as follows. (A) FC/ Sonicated bacteria. (B) FC/ Live bacteria. (C) DRG /Sonicated bacteria. (D) DRG /Live bacteria. Animal numbers for each experiment are indicated after the hashtag. Related to Table 2 . [file Image_8.tif]

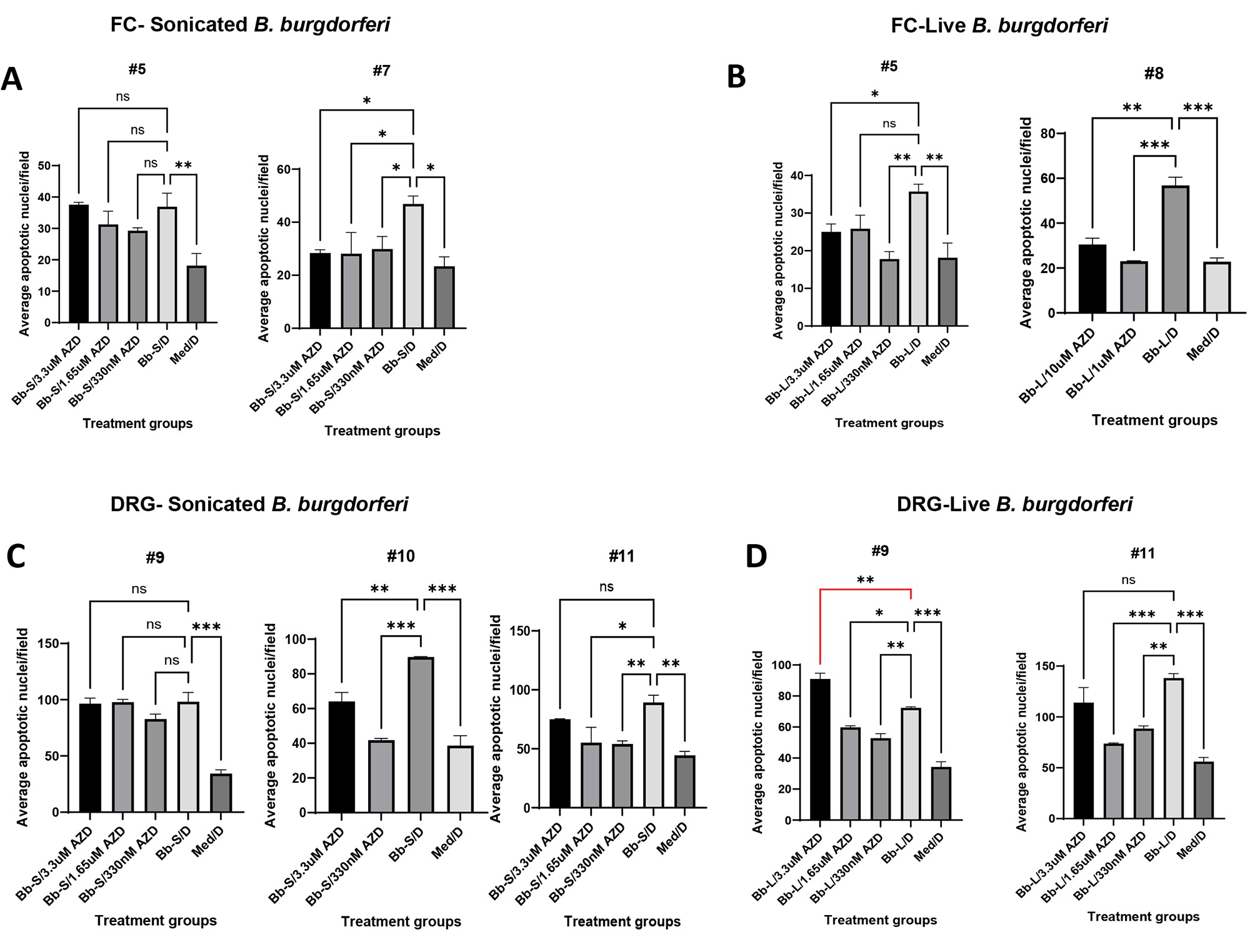

Supplement: Supplementary Figure 9 — Apoptosis levels in the FC and DRG tissues in response to inhibitor AZD4547. Apoptosis levels in response to AZD4547 treatment were measured by the TUNEL assay as described in the methods. (A) FC/ Sonicated bacteria. (B) FC/ Live bacteria. (C) DRG /Sonicated bacteria. (D) DRG /Live bacteria. Bb/S-sonicated B. burgdorferi, Bb/L- Live B. burgdorferi, Med-Medium. D-DMSO, AZD-AZD4547. *p< 0.05; **p< 0.01, *** p< 0.001, **** p < 0. 0001; ns- not significant. All comparisons are with Bb/DMSO group. Black lines and red lines show significantly lower and significantly higher apoptotic levels respectively, compared to Bb/DMSO treatment alone. Medium/DMSO treatment served as a negative control for apoptosis. Animal tissues sourced for each experiment are indicated on each graph. Related to Table 3 . [file Image_9.tif]
